# Prevalence, risk factors, and interventions for obesity in Saudi Arabia: A systematic review

**DOI:** 10.1111/obr.13448

**Published:** 2022-03-26

**Authors:** Victoria Salem, Noara AlHusseini, Habeeb Ibrahim Abdul Razack, Anastasia Naoum, Omar T. Sims, Saleh A. Alqahtani

**Affiliations:** ^1^ Department of Metabolism, Digestion and Reproduction Imperial College London London UK; ^2^ Imperial Centre for Endocrinology Imperial College Healthcare NHS Trust London UK; ^3^ College of Medicine Alfaisal University Riyadh Saudi Arabia; ^4^ College of Medicine King Saud University Riyadh Saudi Arabia; ^5^ Faculty of Medicine and Health Sciences Universiti Putra Malaysia Serdang Malaysia; ^6^ Ritme Consultancy Rotterdam The Netherlands; ^7^ College of Arts and Sciences University of Alabama at Birmingham Birmingham AL USA; ^8^ School of Public Health University of Alabama at Birmingham Birmingham AL USA; ^9^ School of Medicine University of Alabama at Birmingham Birmingham AL USA; ^10^ School of Medicine University of California San Francisco San Francisco CA USA; ^11^ Liver Transplant Centre King Faisal Specialist Hospital & Research Centre Riyadh Saudi Arabia; ^12^ Division of Gastroenterology and Hepatology Johns Hopkins University Baltimore MD USA

**Keywords:** bariatric surgery, body weight loss, lifestyle interventions, obesity, risk factors, Saudi Arabia

## Abstract

Saudi Arabia (SA) has a reported obesity prevalence greater than the global average. Here, we systematically review firstly the prevalence and associated factors (59 studies) and secondly the pharmacological, lifestyle, and surgical interventions for obesity (body mass index, >30 kg/m^2^) in SA (29 studies) between December 2020 and March 2021 in PubMed, Medline, Embase, PsycINFO, and Cochrane. Peer‐reviewed articles in Arabic and English on human adults (aged >18 years) were searched. Among the eight largest studies with sample sizes over 10,000 people, the maximum‐reported obesity prevalence was 35.6%, with notable variations in gender and geographic region. Diet, specifically the move towards Western diet and heavy consumption of sugary beverages, and high levels of inactivity are major contributing factors to obesity. The reported obesity‐risk polymorphisms are not specific. Bariatric surgery is underrepresented, and in general, there is a lack of nationally coordinated studies on weight loss interventions. In particular, the systematic review did not find a body of research on psychological interventions. There is no trial data for the use of GLP‐1 analogs in SA, despite their widespread use. These findings can help policymakers, and practitioners prioritize future research efforts to reduce obesity prevalence in SA.

## 1 INTRODUCTION

Saudi Arabia (SA) notably has an obesity prevalence higher than the global average (35% vs. 13%).^1^ Consequently, SA suffers a higher share of deaths attributable to obesity (18% vs. 8%) and a higher death rate by obesity (116.7 per 100,000 vs. 60 per 100,000).^2^ In response to the immense health and social ramifications of the obesity problem, the SA government is implementing a wide range of policies in its Vision 2030 plan for a healthier population.^3^, ^4^ SA has seen progressive approaches to obesity management, such as early adoption of day case bariatric procedures.^5^, ^6^ However, there are no scholarly publications that effectively aggregate empirical findings from published studies of obesity in adult (aged >18 years) SA population. This review aims to systematically describe the prevalence, associated factors, and interventional (pharmacological, lifestyle, and surgical) approaches to obesity in SA, which will be of broader interest to those looking to understand the global challenges of obesity. We also provide recommendations that will be useful to academics, policymakers, politicians and clinicians in the region.

## 2 METHODOLOGY

**FIGURE 1:**
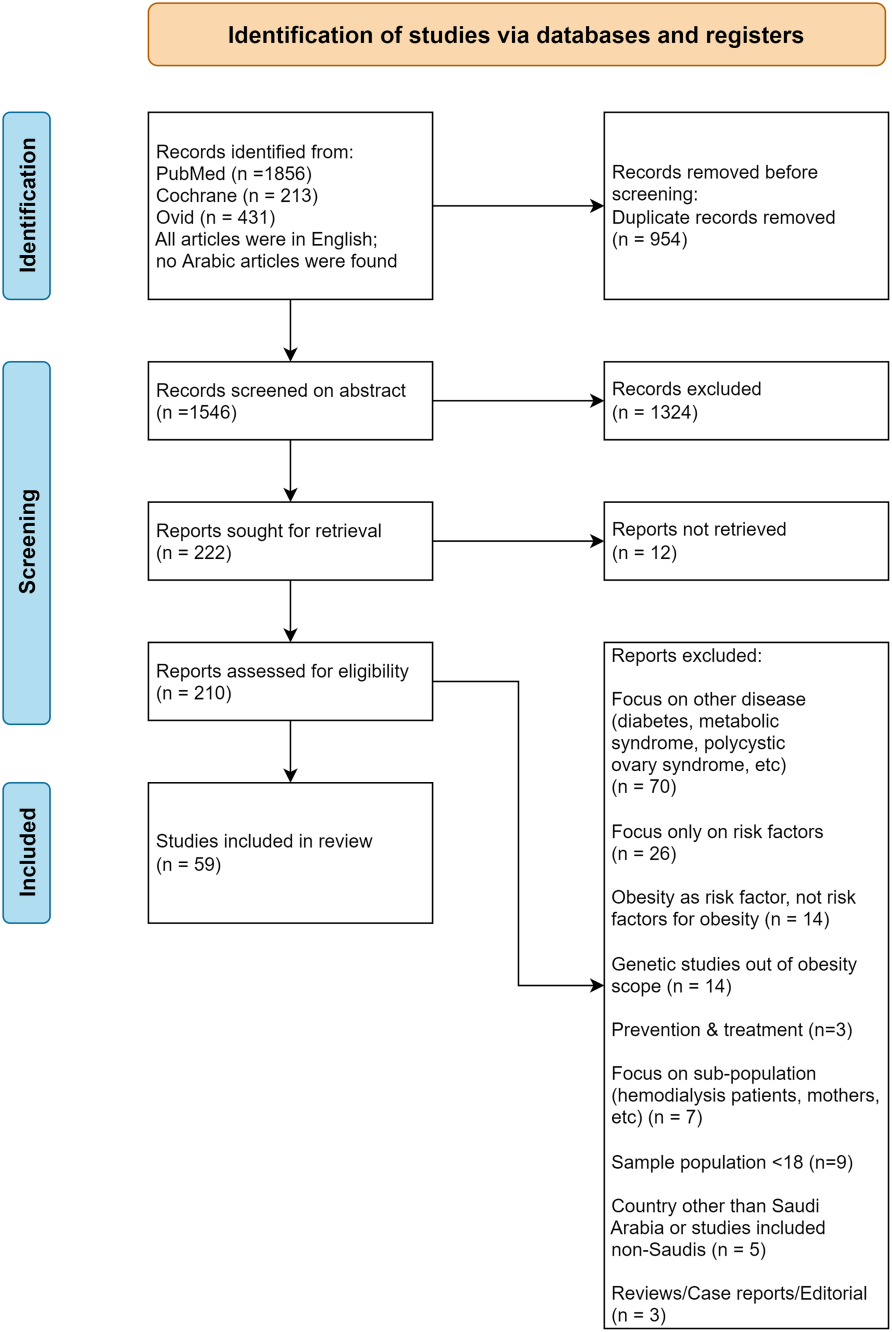
Preferred Reporting Items for Systematic Reviews and Meta‐Analyses (PRISMA) 2020 flow diagram for Research Theme 1

**FIGURE 2:**
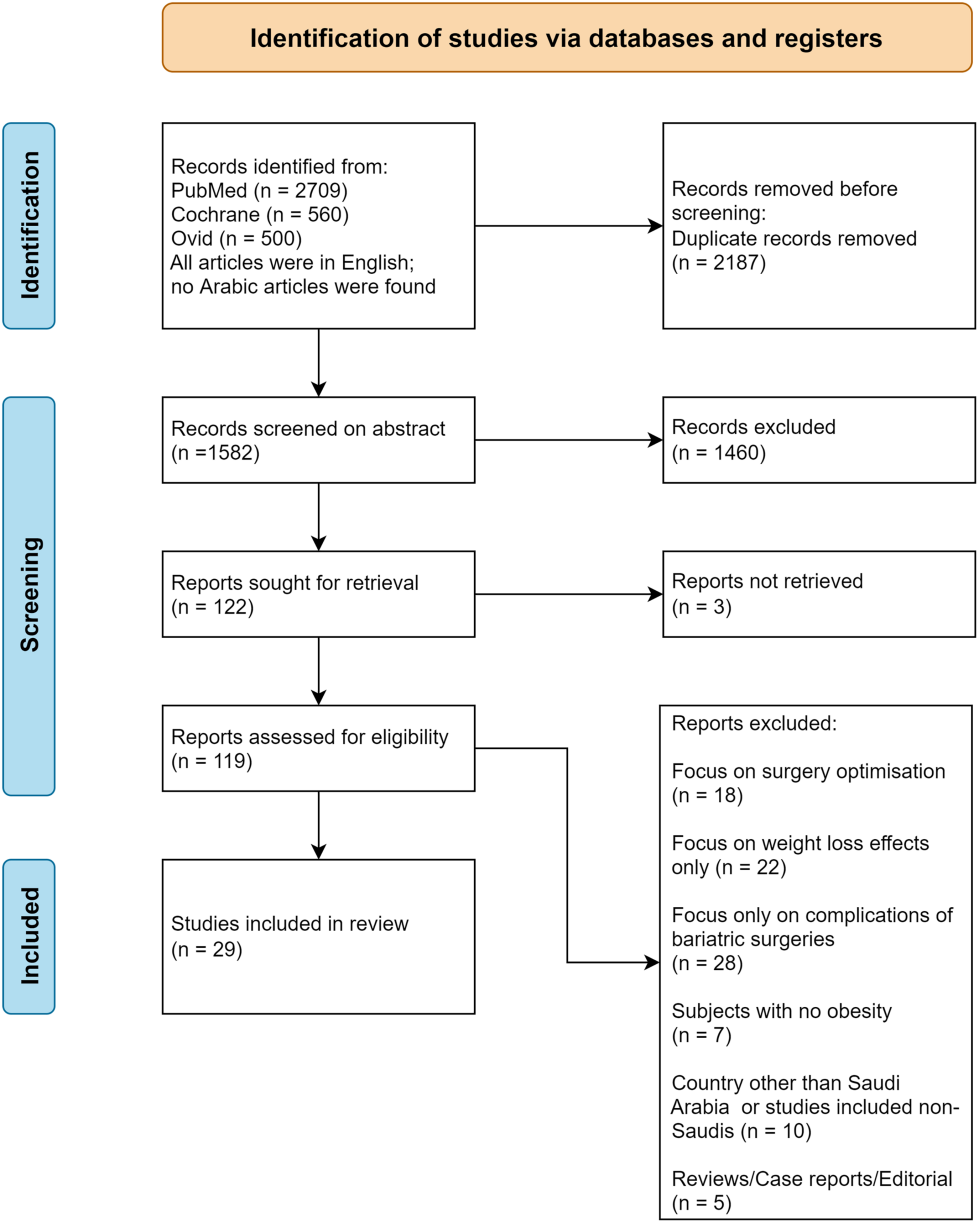
Preferred Reporting Items for Systematic Reviews and Meta‐Analyses (PRISMA) 2020 flow diagram for Research Theme 2

This systematic review was conducted according to the Preferred Reporting Items for Systematic Reviews and Meta‐Analyses (PRISMA) guidelines^7^ and was registered in PROSPERO (CRD42021238386). The PRISMA flowcharts can be found in Figures 1 and 2. Two major research themes were interrogated:

1. What are the demographic, cultural, and epidemiological factors driving obesity in SA?
2. How are effective treatments for obesity currently being delivered to adults with obesity in SA?

Systematic literature searches were carried out between December 2020 and March 2021 for each research theme, applying characteristics defined using the Participant, Intervention, Comparison, Outcome (PICO) system,^8^ with a series of comprehensive electronic searches and filters in PubMed, Medline (Ovid), Embase (Ovid), PsycINFO (Ovid), and Cochrane (see Tables S1–S3). Peer‐reviewed randomized controlled trials (RCTs), cross‐sectional, cohort studies, case–control, and observational were included, but case reports, reviews, and meta‐analyses were not. The search was limited to human adults (aged >18 years) only. Articles published in Arabic and English were searched. One author assessed all studies for risk of bias and then two more authors judged the results. For non‐RCTs, the Cochrane ROBINS‐I^9^ tool was used, whereas, for RCTs, the Cochrane RoB 2 tool^10^ was utilized. For case–control and cohort studies, the Newcastle–Ottawa Scale^11^ was used. Predetermined data fields were agreed upon prior to data extraction into an excel database. We present the results by research theme.

For the first research theme, qualitative and quantitative studies quantifying the prevalence of obesity, defined by body mass index (BMI) >30 kg/m^2^, in SA and those studies showing evidence on the risk factors of obesity, including but not limited to lifestyle, demographics, and genetics, in the SA population were considered. Studies measuring prevalence of obesity in subgroups (pregnant women, hemodialysis patients), prevalence of overweight (defined by BMI 25–30 kg/m^2^) (but not having data on obesity prevalence), or which were conducted in non‐SA locations were excluded.

For the second research theme, qualitative and quantitative studies relating to perceptions, acceptability, efficacy, and safety of treatment interventions (pharmacological, lifestyle, and surgical) for obesity in SA were considered. Glucagon‐like peptide‐1 (GLP‐1) agonist drugs are used to lower glucose levels in patients with diabetes but have been shown to contribute to weight loss, thus are often used in obesity management. Because an initial search for studies restricted to patients in SA using GLP‐1 agonist drugs to treat obesity in the absence of diabetes returned no results, the search criteria were widened to include GLP‐1 agonists licensed for obesity used in the setting of patients with diabetes and obesity. Studies assessing the efficacy, effectiveness, perceptions, and outcomes of bariatric surgeries were also included. Intensive lifestyle intervention studies assessing exercise/physical activity, behavioral changes, and/or dietary regimes with the specified aim of body weight loss (BWL) were also included. Non‐interventional, cross‐sectional studies that only assessed knowledge, awareness, perception, and practices on lifestyle and dietary behaviors to manage obesity were excluded.

## 3 RESULTS

### 3.1 Research Theme 1: Prevalence and risk factors for obesity in SA

The search for Theme 1 yielded 221 articles, which were assessed for eligibility. Of which, 59 eligible studies were included. Summaries of the findings of this theme's search are presented in Table S4.

#### 3.1.1 Prevalence of obesity in SA

Forty‐eight studies reported prevalence estimates of obesity from a variety of general, public, and clinical settings; eight of which had population sizes greater than 10,000 people,^12^, ^13^, ^14^, ^15^, ^16^, ^17^, ^18^, ^19^ among which the maximum‐reported obesity prevalence was 35.6%.^12^ This compares well with 2013 national estimates that showed 24.1% and 33.5% of men and women had obesity, respectively.^20^ Among five studies that classified obesity as defined by WHO,^21^ individuals with severe obesity (BMI > 40 kg/m^2^) constituted 2.1–3.2% of study samples across the reported studies.^14^, ^22^, ^23^, ^24^, ^25^

SA is divided into 13 distinct regions, which differ by population density, affluence, environment, and historical conditions. The prevalence of obesity in SA varied across regions. Althumiri et al. reported that the Eastern Region had the highest prevalence of obesity (BMI > 30 kg/m^2^: 29.4%),^4^ and two large cohort studies^12^, ^13^ agreed that rural residents had a significantly lower prevalence of obesity compared to urban residents (Al‐Nozha et al: 27% vs. 39.7%, *p* < 0.0001).^12^

#### 3.1.2 Age, gender, and marital status

In 18 cross‐sectional studies with over 96,000 participants, increasing age was associated with an increasing prevalence of obesity across all age brackets.^12^, ^13^, ^14^, ^17^, ^18^, ^19^, ^22^, ^23^, ^25^, ^26^, ^27^, ^28^, ^29^, ^30^, ^31^, ^32^, ^33^, ^36^ Women were consistently reported to have a higher rate of obesity in SA,^12^, ^13^, ^30^, ^32^ with one study (*n* = 4758)^32^ reporting twice the rate of obesity in women compared to men. Obesity severity may also be greater in women. The Saudi‐PURE study recruited 2047 participants aged 35–70 from urban and rural households, and although the recruitment mechanisms attempted to avoid bias, the overall incidence of obesity in this cohort was 49.4%. It also revealed that the prevalence of obesity class I (BMI 31–35 kg/m^2^) was higher in men compared with women (39.9% vs. 32.4%), whereas the prevalence of obesity classes II and III (BMI > 35 kg/m^2^) was higher in women compared with men (26.1% vs. 14.5%).^33^ However, one study by Azzeh et al. (*n* = 2548) reported higher waist circumference and visceral fat in men.^22^ Being married, especially for women, was associated with an increased risk of obesity in 8 cross‐sectional studies with more than 38,000 participants.^14^, ^19^, ^25^, ^27^, ^28^, ^29^, ^30^, ^34^

#### 3.1.3 Education and income

Education level and its association with obesity rates were examined in 11 cross‐sectional studies with over 51,000 participants combined.^13^, ^14^, ^17^, ^18^, ^19^, ^26^, ^27^, ^30^, ^31^, ^34^, ^35^ The study findings were controlled for confounding effects by multivariate statistical analyses. In three studies, education was not related (no statistical significance) to obesity rates.^27^, ^30^, ^31^ In six studies (*n* = 31,201), the prevalence of obesity was higher among those with low educational attainment.^13^, ^14^, ^26^, ^34^, ^35^, ^36^ However, the relationship might not be linear; in four studies (*n* = 32,111), education beyond high school level was significantly associated with increased obesity risk.^17^, ^18^, ^19^, ^29^

The findings on the association between income and obesity are conflicting, which again may represent a nonlinear “u‐shaped” association or differences in study design. In four cross‐sectional studies (*n* = 28,065), the prevalence of obesity was highest among high‐income participants,^13^, ^17^, ^34^, ^37^ whereas in two other studies (*n* = 660), the prevalence of obesity was highest among the low‐income group.^29^, ^35^

Of note, in a cross‐sectional study with over 3000 participants, the relationship between educational level and obesity based on income was explored; in the high‐income group, the odds of obesity among those with only middle or high school education was 2.15 (95% CI: 1.48–3.11) compared with 1.24 (95% CI: 1.01–1.50) in those with a college degree or higher. Further, participants with an elementary school education level were 2.12 (95% CI: 1.32–3.39) times more likely to have obesity in the low‐income group compared with 4.29 (95% CI: 2.35–7.84) in the higher income group.^34^

These findings support the notion of a nonlinear interaction between income and educational level as predictors of obesity.

#### 3.1.4 Obesity‐related comorbidities

Numerous studies confirm the expected association of obesity in populations with chronic diseases, including diabetes, hypertension, and dyslipidemia. The results of all these studies indicate that those with chronic diseases are more likely to have obesity.^14^, ^15^, ^19^, ^24^, ^27^, ^31^, ^33^

For example, the odds of obesity were 1.6–3.72 among those with dyslipidemia or hyperlipidemia.^24^, ^27^ However, although all the studies adjusted for confounding variables with statistical regressions, reverse causality has not been well explored.

#### 3.1.5 Sleep hygiene

In a cohort study with 2686 participants, people sleeping more than 8 h a night compared with those sleeping 7 h were 1.54 times more likely to be affected by obesity, an association that survived correction for multiple confounders.^36^

#### 3.1.6 Psychosocial factors

In two studies (*n* = 1,199) on college students, participants with obesity had more siblings in the family and more parents affected by obesity compared to participants with no obesity.^24^, ^31^ For example, the odds of developing obesity was reported as 10.99 if the index patient's father had obesity.^24^ However, the studies did not explain if this relationship was due to environmental or genetic factors. In a case–control study by Mattoo et al., parental negligence in feeding behavior (false perception of obesity, not asserting feeding control, not providing nutritious meals, breakfast skipping, eating outside, not instilling proper feeding practices) was found to be a predictor of obesity, although it was not clear to what extent this was common or an important driver in this country in general. Adults with obesity were also more likely to have had mothers who stopped breastfeeding early and introduced formula milk.^35^

Intimate partner violence, child abuse, depression, and posttraumatic stress disorder symptoms are related to obesity in SA like many other countries.^16^, ^38^ In a cross‐sectional, nationwide study of 10,156 participants, adverse childhood effects (ACEs)—emotional, physical, sexual abuse or neglect, or any other stressors including domestic violence, drugs or alcohol abuse, mental illness, or criminal activities—were found to increase the risk of obesity among adults. In this unbiased observational study, 39% had experienced three or more ACEs.^38^ Women who suffered four or more ACEs were 2.5 times more likely to develop obesity (95%, CI: 1.7–3.7), whereas the odds were 2.2 for men (95%, CI: 1.2–3.5).^16^ These results are comparable with the incidence of ACE and its impact on obesity risk in the U.S. and European studies.^39^, ^40^

#### 3.1.7 Dietary habits

Twenty‐one studies focused on dietary habits; however, only a few are large, population‐based studies. The majority of the studies focused on young adults. Among King Saud University students (*n* = 312), caloric intake was mainly derived from carbohydrates (72%), and protein sources were largely of animal origin.^37^ In another study, the majority of respondents (70.5%) were aware of the importance of dietary fiber for controlling obesity, but there was a disconnect in translating this knowledge into dietary practices due to the perception that fiber‐rich food is expensive and unpalatable. Respondents in that study with the highest fiber consumption had a lower prevalence of obesity (*p* = 0.03).^41^

Five studies revealed suboptimal fruits and vegetables dietary consumption in SA.^14^, ^32^, ^42^, ^43^, ^44^ One study accessing the Saudi Health Information Survey among 13 health regions in SA (*n* = 10,735) reported that over 81% of males and females consumed less than three servings of fruits and vegetables per day.^14^ Another national cross‐sectional study among five regions (Central, East, West, North, and South) of SA (*n* = 4883) revealed that less than 6% of participants consumed five or more servings of fruits and vegetables daily.^32^

Seven studies concluded suboptimal dietary habits among participants, including excessive consumption of fast food, fried foods, french fries/potato chips, sweets, frequent snacks, energy drinks, and sugary beverages among adults in SA.^43^, ^44^, ^45^, ^46^, ^47^, ^48^, ^49^ There was a concentration of studies highlighting unhealthy eating behaviors in young adults. A study among students in Jizan (*n* = 416) confirmed that the majority of participants with obesity (54.8%) ate snacks with carbonated beverages while watching TV daily. There was also a significant association between BMI and food consumption setting (e.g., dining on a table or the floor *p* < 0.01).^50^ A study among students in Rabigh revealed 57% ate fast food weekly, and 43% consumed soft or energy drinks more than once per day.^48^ In Dammam city, over a third of male university students consumed fast food and sugary beverages 6–10 times per week (35.8%).^43^ Another study conducted among dental students in Riyadh (*n* = 270) showed that the consumption of carbonated beverages was higher among the group affected by obesity (21.4%).^46^

#### 3.1.8 Physical activity

We found 16 studies related to physical activity in our search. Four national studies found high inactivity levels among participants in SA.^14^, ^19^, ^32^, ^51^ The Saudi Health Interview Survey (*n* = 10,735) found that 46.5% of participants did not engage in any regular physical activity at all and 50% spent over 3 h daily watching TV.^19^ Accordingly, the risk of obesity was lower among active males compared with inactive males.^14^ Data from the National Epidemiologic Health Survey between 1995 and 2000 (*n* = 17,395) found that the prevalence of inactivity was high, with no significant difference in the physical inactivity levels between urban and rural residents.^51^ Another national study (*n* = 4883) reported high inactivity levels as participants spent 4.5 h per day sedentary, and 60% of males and 73% of females failed to meet moderate physical activity level criteria.^32^

#### 3.1.9 Genetic factors

A total of 11 studies identified genetic polymorphisms associated with obesity among adults in SA.^52^, ^53^, ^54^, ^55^, ^56^, ^57^, ^58^, ^59^, ^60^, ^61^, ^62^ Most studies were case–control studies with comparisons between adults with and without obesity. Five of the 11 studies were conducted at King Saud University, with sample sizes ranging from 204 to 492 participants.^54^, ^55^, ^56^, ^57^, ^58^ Polymorphisms that independently increased the odds of obesity were found in the genes for uncoupling protein 1 (rs1800592 [OR = 1.52, CI: 1.10–2.08, *p* = 0.009]); the fat‐mass and obesity‐associated gene (rs1421085 [OR = 1.56, CI: 1.04–2.34, *p* = 0.03]), and melanocortin‐4 receptor (rs17782313 [OR = 1.72, CI: 1.02–2.89, *p* = 0.038]). There is no convincing data to suggest that these or any other obesity‐predictive polymorphisms are enriched in the Saudi population.^59^, ^60^, ^61^, ^62^

### 3.2 Research Theme 2: Treatment

The search for Theme 2 yielded 122 articles, which were assessed for eligibility. Of which, 29 studies were included. Summaries of the results of this research theme can be found in Table S5. The risk of bias assessment indicated low risk or some concerns for all studies except one.^63^ The results are not analyzed further in the paper but are included in the number of studies on bariatric surgery results. The results of risk of bias analysis can be found in Tables S6 and S7.

#### 3.2.1 Treatment of obesity with intensive lifestyle interventions

Intensive lifestyle interventions are the cornerstone of the SA clinical practice guideline for the management of obesity.^64^ Only four high‐quality prospective or randomized lifestyle intervention studies were conducted in SA.^65^, ^66^, ^67^, ^68^ This totaled 620 participants, of whom 260 were exposed to an intensive arm. Of note, three of the four studies recruited women only,^65^, ^66^, ^67^ and in total, 408 (61%) of all participants across the studies were female. A primary outcome target of BWL > 5% was reported in two studies. Other outcome measures included quality of life and surrogate markers of metabolic inflammation.

In brief, the interventions were exercise only (aerobic or resistance, three 40‐min sessions a week for 3 months) or “intensive” lifestyle advice. The latter varied to include low‐fat diet (not calorie counted) with the provision of a pedometer; written dietary advice as per the Diabetes Prevention program^69^ with behavioral techniques; or 1200 kcal/day dietary restriction with regular 40‐min aerobic exercise sessions. Of note, the number of encounters with health care professionals during “intensive” programs was poorly recorded. Comparator groups of “standard” advice ranged from no intervention at all to a single education session with a health care professional and written materials.

Weight loss data were available for 199 patients in intensive arms. The average weight loss achieved was 4.1% of starting body weight. This compares favorably with the weight‐loss results of other published intensive lifestyle interventions,^69^, ^70^ although it does not meet the >5% weight loss that is established to best correlate with risk reduction in obesity‐associated comorbidity, which is needed to reduce the risk of obesity‐related illness and induce remission of established co‐morbidities.^71^, ^72^, ^73^ Dropout rates (taken from the intensive groups) averaged 16%, suggesting good engagement levels.

#### 3.2.2 Pharmacological treatments for obesity

GLP‐1 agonists were developed as a glucose‐lowering treatment in type 2 diabetes; however, as a class, they are highly efficacious at lowering body weight, leading to FDA approval for the treatment of obesity in the absence of diabetes.^74^, ^75^ SA holds the highest market share of GLP‐1 agonists in the Middle East and Africa,^76^ and these agents are frequently prescribed for the treatment of obesity in patients with and without diabetes. Nevertheless, to date, there is no study of GLP‐1 agonist usage in the population with obesity‐only (without diabetes) in SA. Extending the search to look at all registered clinical trials for GLP‐1 drugs did not find contributing centers in SA.

Two studies were identified here using real‐world usage of GLP‐1 analogs in participants with obesity and type 2 diabetes that includes weight loss data.^77^, ^78^ In the first study, liraglutide treatment mitigated the expected weight gain in this cohort who were also being treated with insulin, and 12‐month weight change was nonsignificantly different from baseline,^77^ and in the second study, 12‐week weight loss in treatment naïve patients averaged 3.9 kg (4.2%). This response to liraglutide mirrors other real‐world studies,^79^ but of note, the dose was lower than that currently advocated for obesity, and newer formulations of GLP‐1R agonists are likely to be even more potent. Neither study documented side effects. Thus, in summary, trial data for efficacy and tolerability of GLP‐1 analogs in the SA population with obesity are lacking.

#### 3.2.3 Bariatric surgery provision

Healthcare in SA comprises a hybrid of government care financed and run by the Ministry of Health (MOH) as well as self‐pay or insurance‐funded private provision.^80^ Obesity treatments, including bariatric surgery, are available in the public and the private healthcare sectors. In a survey conducted by the Asia‐Pacific Metabolic and Bariatric Surgery Society,^81^ 70 bariatric surgeons working across 30 institutions were identified in SA. The Saudi Clinical Practice Guidelines for the Management of Obesity^64^ and the SA Society for Metabolic and Bariatric Surgery^82^ both state a BMI of 40 kg/m^2^ with or without an obesity‐related comorbidity or 35 kg/m^2^ in the presence of obesity‐associated illness as an indication for bariatric surgery. This is in line with BMI eligibility criteria for bariatric surgery in other countries such as the United Kingdom.^83^

#### 3.2.4 Bariatric surgery uptake

There were over 27,000 bariatric operations in SA in 2019,^84^ one of the highest per capita procedure rates in the world. Of note, this only captures public sector data, and we estimate that a similar number of operations were also carried out in the private sector. In 2018, three SA centers submitted their bariatric surgery records to the International Federation for the Surgery of Obesity and Metabolic Disorders (IFSO), totaling 4231 procedures.^85^ However, this only represents 10% of all centers carrying out bariatric operations in SA,^81^ although this still constitutes 30% of cases that were submitted to IFSO across six Gulf Cooperation Council member states. As a result, there is reporting bias in bariatric surgery metrics for SA. For example, according to the IFSO dataset, 100% of bariatric procedures carried out in SA are sleeve gastrectomy, but this is not in accordance with the available data.^84^ Furthermore, SA has the lowest median age for primary procedure among all of the countries reported in the IFSO dataset, at 28.5 years. However, the center that submitted most of the SA data to IFSO specializes in child and adolescent surgery. Between 2014 and 2018, IFSO data reported that 67% of bariatric surgery recipients in SA were female, which sits about halfway across the range of countries reporting gender uptake (51–94%). In SA, just under 20% of patients reported to the database had preexisting type 2 diabetes. This is lower than the national prevalence of about 25% (which is the seventh highest in the world).^86^, ^87^

#### 3.2.5 Perceptions of bariatric surgery

Five cross‐sectional studies were identified looking at public perceptions of obesity and bariatric surgery in SA.^88^, ^89^, ^90^, ^91^, ^92^ A total of 3131 people were surveyed or interviewed, of which 64% were women. Across three studies, 26% of respondents thought that bariatric surgery was primarily a cosmetic procedure.^88^, ^91^, ^92^ In the largest cohort, 40% said they would not want bariatric surgery, although the mean BMI in this cohort was only 28 kg/m^2^.^88^ This may be linked to high levels of perceived risk with these procedures.^89^, ^92^ However, in the only study that solely interviewed people with obesity, the seven female participants indicated much higher rates of bariatric surgery acceptance among people living with obesity.^90^

#### 3.2.6 Complications of bariatric surgery

The Obesity Surgery Mortality Risk Score stratifies patients undergoing bariatric surgery into three categories, depending on the number of risk factors they possess. This is very poorly recorded for the IFSO SA datasets (<2% return rate).^85^ It is also unclear whether day case procedures are more commonly performed in SA (i.e., whether the operation is more commonly performed in lower‐risk patient groups).

We identified nine papers, comprising 1078 patients, and a total of 195 reported complications of varying severity. Mortality rates were published. Of these papers, one was a case series, one cross‐sectional study, one prospective observational study, and six were retrospective audits.^93^, ^94^, ^95^, ^96^, ^97^, ^98^, ^99^, ^100^, ^101^ Individual case reports were excluded. The studies included one type of bariatric procedure^93^, ^95^, ^98^, ^99^, ^101^ and compared complications across up to five different bariatric interventions.^96^ There were four studies with no denominator.^93^, ^95^, ^97^, ^99^ These described 14 cases of acute pancreatitis after post‐intragastric balloon,^93^, ^95^ 13 cases of acute axonal polyneuropathy,^98^ and 42 cases of postoperative leak after sleeve gastrectomy.^99^

Collating data where a denominator of observed cases was provided totaled 1009 bariatric procedures. These studies reported 3% incidence of neurological complications following bariatric surgeries; of which, 60% were peripheral neuropathy,^94^ 6.6% thoracic complications (75% of which were pleural effusions),^96^ 35% rate of new‐onset gastro‐oesophageal reflux disease post‐sleeve gastrectomy,^98^ 8% readmission rate after surgery—most commonly for pain management—and finally, 14% incidence of new nutritional deficiency after bariatric surgery (restricted to B12 and folate and of note nutritional deficiencies were actually more common and needed to be treated preoperatively).^101^

#### 3.2.7 Outcomes of bariatric surgery

Ten studies were identified reporting weight loss outcomes with bariatric surgery in SA,^101^, ^102^, ^103^, ^104^, ^105^, ^106^, ^107^, ^108^, ^109^, ^110^ none of which compared with a lifestyle or pharmacologically treated group. In total, 1937 surgeries were reported; of which, 79% were performed on women, and the average age was 34.6 years. Average BMI at surgery was 37.9 (*n* = 1597), and average body weight at surgery was 123.7 kg (*n* = 770). Three studies report textured information on comorbidities at baseline for 342 patients.^106^, ^107^, ^110^ Among these, the most common obesity‐related comorbidity were, in order of frequency, obstructive sleep apnoea (35%), hypertension (20%), and type 2 diabetes mellitus (13%).

Average weight loss in kilograms (*n* = 867 reported, calculated for 6‐month postoperative or as close as possible to that time point) was 33.6 kg, and %BWL was 18% (*n* = 1592). This discrepancy between actual weight loss and percentage weight loss is weighted by two studies that report large average weight losses of 52 and 40.3 kg, respectively.^107^, ^108^ It was not possible to stratify weight loss by gender. It is increasingly accepted that bariatric procedures should be seen in terms of metabolic rather than weight loss surgery.^111^ However, only two SA studies specifically commented on postoperative improvements in obesity‐associated comorbidities,^102^, ^104^ although not in fine detail.

## 4 DISCUSSION

This systematic review identifies risk factors associated with obesity in SA and summarizes clinical interventions that have been trialed and reported in the country. Many of the associations reviewed here are well documented globally and are equally pertinent to SA. However, it is also worthwhile venturing into some interpretations as to why obesity levels are particularly high in SA.

There is a disparity between the genders in terms of obesity rates in SA compared with other countries; specifically, married, older women have high levels of obesity in this country. The reasons for this are likely to be complex. In 2019, the Saudi MOH released the latest results of the Saudi data from the World Health Organisation's World Health Survey (SAWHS). This reported 20% of people in SA had obesity, with a higher percentage among females (21%), urban areas (21%), and those without formal education (29%). Obesity also increased with age from 10% in the age group 18–29 to 20% in 70–79, before falling to 22% among the elderly above 80 years.^112^ Much more research is required to understand the cultural and emotional drivers for weight gain and how best to overcome them. Physical inactivity is high in SA. SAWHS 2019 showed insufficient physical activity among 80% of the population that reportedly increased by age, residing in rural areas, and having low educational attainment.^112^ SA's vast economic growth has led to rapid developments in living standards and urbanization, which increased reliance on cars. The extremely hot weather also prevents outdoor activities.^113^ Laws have been passed allowing the introduction of physical activity classes in female public schools via the Vision 2030 and the National Transformation Program.^3^ We would urge a preemptive evaluation of these initiatives to identify populations with common risk factors who are being positively impacted by the new policies and to identify iterative public health policies that may be needed to reach and impact populations who are without noticeable improvements.

Genetic studies in the region are currently limited but must be an important focus for future studies as this may inform intervention approaches. For example, in a recently published systemic review, there is evidence that some of the polymorphism described in studies conducted in SA may be enriched in the wider Arab world.^114^ Dietary studies in SA also, in general, lack clarity and the ability to discriminate local practices and customs that may be particular drivers of weight gain. During the past three decades, SA has witnessed a major shift from its traditional diet to the Western diet, particularly high in animal fats and refined sugars.^41^, ^42^ SAWHS 2019 also identified insufficient intake of fruits and vegetables among 93% of participants.^112^ Young, urban adults, in particular, seem to have fallen prey to the abundance of convenient and highly palatable nontraditional food options. In the global analysis of daily calories sold per capita from all sugar‐sweetened beverages, the top six countries were Chile, Mexico, the United States, SA, Argentina, and Germany.^115^ Interestingly, the 50% *ad valorem* tax on carbonated beverages introduced by the Saudi government in 2017 did reduce the volume sale of carbonated drinks by 35%.^115^ Moreover, as part of the Saudi Vision 2030,^3^ the Saudi Food and Drug Authority has developed the “Healthy Food Strategy,” which incorporates a series of nutritional reforms and educational campaigns to enhance healthy lifestyles and reduce the intake of sugar, salt, and fatty acids.^116^ However, more evidence is required to assess the long‐term effects, to what extent sugary drinks are still major drivers of poor health, and whether such interventions would work on other foodstuffs.

In the second part of the systematic review, we looked at intensive lifestyle interventions, pharmacological agents, and bariatric surgery that have been reported to treat obesity in SA. These approaches are consistent with guidelines on the prevention and management of obesity that were published by SA's MOH but with one exception. The systematic review did not find a body of research that commonly used psychological interventions and strategies in weight management for obesity. Guidelines from SA's MOH specify that psychological interventions should be integrated into weight management efforts to decrease dietary energy intake, increase physical activity, and decrease sedentary behaviors.^117^ Psychological interventions could include stimulus control (i.e., means to recognize and avoid triggers that prompt unplanned eating), cognitive restructuring (i.e., modifying unhelpful thoughts and thinking patterns), goal setting, assertiveness training (i.e., stress and anger management to improve coping skills), and strategies for dealing with weight regain. Psychological interventions have the potential to facilitate uptake of lifestyle interventions, to improve uptake and adherent use of pharmacological agents, and to mitigate noncompliance with dietary recommendations that often lead to weight regain or failure to reach the appropriate weight after bariatric surgery. Without consistent use and inclusion of psychological interventions for weight management or treatment of obesity in public health and clinical settings, the impact of lifestyle advice, pharmacological agents, and bariatric surgery may be futile or limited.

Finally, this systematic review also highlighted further areas where coordinated research efforts could be improved. Given that SA has many centers of excellence for bariatric surgery, there is a clear need now for improved coordination in terms of a national database for audit, as well as building opportunities for multicenter trials. The figures suggesting that patients with diabetes are relatively underrepresented in those undergoing bariatric surgery in SA points to the possibility of expanding the concept and role for early “metabolic” surgery in this arena. Finally, we note that despite its high prevalence of obesity and diabetes, SA is underrepresented in terms of contributing patients and centers to multinational trials.

This systematic review has notable limitations and strengths. We used leading scholarly databases and exhaustive combinations of search terms to identify relevant literature published in English and Arabic; however, our search did not identify any articles in Arabic. Notwithstanding, literature available in English was thoroughly reviewed, and two types of bias assessments were conducted (contingent on study design). The PICO and PRISMA guidelines were rigorously followed to shape and to guide the systematic review, and studies with heterogeneous study designs were included. To our knowledge, this is the first systematic review that has been conducted with a focus on the prevalence and determinants of obesity among adults in SA.

## 5 CONCLUSION

In SA, the prevalence of obesity in SA is greater than the global prevalence of obesity, and risk factors are multifactorial. Three intervention approaches are commonly used to treat obesity, while psychological interventions are underutilized. Policymakers, public health professionals, and practitioners engaged in public health and clinical management of obesity are encouraged to use findings to expedite nationwide reductions in the prevalence of obesity in SA.

## CONFLICT OF INTEREST

Authors declare no conflicts of interest.

## AUTHOR CONTRIBUTIONS

SA conceived the study. HI and AN conducted the systematic literature search in scholarly databases. AN performed the risk of bias analysis. VS and OS checked the eligibility of included studies and results of risk of bias analysis. HI, AN, and NA extracted data from selected studies and prepared summary table. HI, AN, NA, OS, VS, and SA wrote the manuscript. VS and SA reviewed and substantially revised the manuscript content. All authors reviewed and approved the final version of the manuscript.

## Supporting information


**Table S1:** Search strategy for Pubmed
**Table S2:** Search strategy for Ovid
**Table S3:** Search strategy for Cochrane
**Table S4:** Results of systematic literature review for research theme 1
**Table S5:** Results of systematic literature review for research theme 2
**Table S6:** Risk of bias assessment of RCTs and case–control studies for research theme 2
**Table S7:** Risk of bias assessment of observational studies for research theme 2
