# Supplementary material for: Prevalence, risk factors, and interventions for obesity in Saudi Arabia: A systematic review
Source: Obes Rev. 2022 Mar 26;23(7):e13448. doi: 10.1111/obr.13448 (PMC9287009; doi:10.1111/obr.13448)
Supplement: Supplementary file 1 — Table S1: Search strategy for Pubmed Table S2: Search strategy for Ovid Table S3: Search strategy for Cochrane Table S4: Results of systematic literature review for research theme 1 Table S5: Results of systematic literature review for research theme 2 Table S6: Risk of bias assessment of RCTs and case–control studies for research theme 2 Table S7: Risk of bias assessment of observational studies for research theme 2 [file OBR-23-0-s001.docx]

**Supplementary File**

**Prevalence, Risk Factors, and Interventions for Obesity in Saudi Arabia: A Systematic Review**

Victoria Salem,^1,2^ Noara AlHusseini,^3^ Habeeb Ibrahim Abdul Razack,^4,5^ Anastasia Naoum,^6^ Omar T. Sims,^7,8,9,10^ Saleh A. Alqahtani ^11,12^*

^1^ Department of Metabolism, Digestion and Reproduction, Imperial College London, London, UK

^2^ Imperial Centre for Endocrinology, Imperial College Healthcare NHS Trust, London, UK

^3^ College of Medicine, Alfaisal University, Riyadh, Saudi Arabia

^4^ College of Medicine, King Saud University, Riyadh, Saudi Arabia

^5^ Faculty of Medicine & Health Sciences, Universiti Putra Malaysia, Serdang, Selangor, Malaysia

^6^ Ritme Consultancy, Rotterdam, The Netherlands

^7^ College of Arts and Sciences, University of Alabama at Birmingham, Birmingham, AL, USA

^8^ School of Public Health, University of Alabama at Birmingham, Birmingham, AL, USA

^9^ School of Medicine, University of Alabama at Birmingham, Birmingham, AL, USA

^10^ School of Medicine, University of California San Francisco, San Francisco, CA, USA

^11^ Liver Transplant Centre, King Faisal Specialist Hospital & Research Centre, Riyadh, Saudi Arabia

^12^ Division of Gastroenterology and Hepatology, Johns Hopkins University, Baltimore, MD, USA

Corresponding Author:

Dr Saleh A. Alqahtani M.D.,

Liver Transplant Centre, King Faisal Specialist Hospital & Research Centre,

Riyadh 12713, Saudi Arabia. Email: [salalqahtani@kfshrc.edu.sa](mailto:salalqahtani@kfshrc.edu.sa)

Contents:

Table S1: Search strategy for Pubmed

Table S2: Search strategy for Ovid

Table S3: Search strategy for Cochrane

Table S4: Results of systematic literature review for research theme 1

Table S5: Results of systematic literature review for research theme 2

Table S6: Risk of bias assessment of RCTs and case-control studies for research theme 2

Table S7: Risk of bias assessment of observational studies for research theme 2

Table S1. Search strategy for Pubmed

| A) Research Theme 1: What are the demographic, cultural and epidemiological factors driving obesity in SA? |
| --- |
| 1. "obeses"[All Fields] OR "obesity"[MeSH Terms] OR "obesity"[All Fields] OR "obese"[All Fields] OR "obesities"[All Fields] OR "obesity s"[All Fields] OR ("overweight"[MeSH Terms] OR "overweight"[All Fields] OR "overweighted"[All Fields] OR "overweightness"[All Fields] OR "overweights"[All Fields]) OR ("metabolic syndrome"[MeSH Terms] OR ("metabolic"[All Fields] AND "syndrome"[All Fields]) OR "metabolic syndrome"[All Fields]) OR "waist-circumference"[All Fields] OR "BMI"[All Fields] 2. "saudi arabia"[MeSH Terms] OR ("saudi"[All Fields] AND "arabia"[All Fields]) OR "saudi arabia"[All Fields] OR "Gulf"[All Fields] OR "KSA"[All Fields] 3. ("obeses"[All Fields] OR "obesity"[MeSH Terms] OR "obesity"[All Fields] OR "obese"[All Fields] OR "obesities"[All Fields] OR "obesity s"[All Fields] OR ("overweight"[MeSH Terms] OR "overweight"[All Fields] OR "overweighted"[All Fields] OR "overweightness"[All Fields] OR "overweights"[All Fields]) OR ("metabolic syndrome"[MeSH Terms] OR ("metabolic"[All Fields] AND "syndrome"[All Fields]) OR "metabolic syndrome"[All Fields]) OR "waist-circumference"[All Fields] OR "BMI"[All Fields]) AND ("hasabstract"[All Fields] AND ("pubmed books"[Filter] OR "case reports"[Publication Type] OR "clinical study"[Publication Type] OR "clinical trial"[Publication Type] OR "clinical trial, phase i"[Publication Type] OR "clinical trial, phase ii"[Publication Type] OR "clinical trial, phase iii"[Publication Type] OR "clinical trial, phase iv"[Publication Type] OR "comparative study"[Publication Type] OR "controlled clinical trial"[Publication Type] OR "government publication"[Publication Type] OR "historical article"[Publication Type] OR "introductory journal article"[Publication Type] OR "journal article"[Publication Type] OR "multicenter study"[Publication Type] OR "observational study"[Publication Type] OR "overall"[Publication Type] OR "pragmatic clinical trial"[Publication Type] OR "randomized controlled trial"[Publication Type] OR "technical report"[Publication Type] OR "twin study"[Publication Type] OR "validation study"[Publication Type]) AND "humans"[MeSH Terms] AND 1950/01/01:2020/12/31[Date - Publication] AND ("adult"[MeSH Terms] OR "young adult"[MeSH Terms] OR "adult"[MeSH Terms:noexp] OR ("middle aged"[MeSH Terms] OR "aged"[MeSH Terms]) OR "middle aged"[MeSH Terms] OR "aged"[MeSH Terms] OR "aged, 80 and over"[MeSH Terms])) AND (("saudi arabia"[MeSH Terms] OR ("saudi"[All Fields] AND "arabia"[All Fields]) OR "saudi arabia"[All Fields] OR "Gulf"[All Fields] OR "KSA"[All Fields]) AND ("hasabstract"[All Fields] AND ("pubmed books"[Filter] OR "case reports"[Publication Type] OR "clinical study"[Publication Type] OR "clinical trial"[Publication Type] OR "clinical trial, phase i"[Publication Type] OR "clinical trial, phase ii"[Publication Type] OR "clinical trial, phase iii"[Publication Type] OR "clinical trial, phase iv"[Publication Type] OR "comparative study"[Publication Type] OR "controlled clinical trial"[Publication Type] OR "government publication"[Publication Type] OR "historical article"[Publication Type] OR "introductory journal article"[Publication Type] OR "journal article"[Publication Type] OR "multicenter study"[Publication Type] OR "observational study"[Publication Type] OR "overall"[Publication Type] OR "pragmatic clinical trial"[Publication Type] OR "randomized controlled trial"[Publication Type] OR "technical report"[Publication Type] OR "twin study"[Publication Type] OR "validation study"[Publication Type]) AND "humans"[MeSH Terms] AND 1950/01/01:2020/12/31[Date - Publication] AND ("adult"[MeSH Terms] OR "young adult"[MeSH Terms] OR "adult"[MeSH Terms:noexp] OR ("middle aged"[MeSH Terms] OR "aged"[MeSH Terms]) OR "middle aged"[MeSH Terms] OR "aged"[MeSH Terms] OR "aged, 80 and over"[MeSH Terms]))) 4. "risk*"[All Fields] OR "epidem*"[All Fields] OR "behav*"[All Fields] OR "habit*"[All Fields] OR ("life style"[MeSH Terms] OR ("life"[All Fields] AND "style"[All Fields]) OR "life style"[All Fields] OR "lifestyle"[All Fields] OR "lifestyles"[All Fields]) OR ("epidemiology"[MeSH Subheading] OR "epidemiology"[All Fields] OR "prevalence"[All Fields] OR "prevalence"[MeSH Terms] OR "prevalance"[All Fields] OR "prevalences"[All Fields] OR "prevalence s"[All Fields] OR "prevalent"[All Fields] OR "prevalently"[All Fields] OR "prevalents"[All Fields]) 5. ("obeses"[All Fields] OR "obesity"[MeSH Terms] OR "obesity"[All Fields] OR "obese"[All Fields] OR "obesities"[All Fields] OR "obesity s"[All Fields] OR ("overweight"[MeSH Terms] OR "overweight"[All Fields] OR "overweighted"[All Fields] OR "overweightness"[All Fields] OR "overweights"[All Fields]) OR ("metabolic syndrome"[MeSH Terms] OR ("metabolic"[All Fields] AND "syndrome"[All Fields]) OR "metabolic syndrome"[All Fields]) OR "waist-circumference"[All Fields] OR "BMI"[All Fields]) AND ("hasabstract"[All Fields] AND ("pubmed books"[Filter] OR "case reports"[Publication Type] OR "clinical study"[Publication Type] OR "clinical trial"[Publication Type] OR "clinical trial, phase i"[Publication Type] OR "clinical trial, phase ii"[Publication Type] OR "clinical trial, phase iii"[Publication Type] OR "clinical trial, phase iv"[Publication Type] OR "comparative study"[Publication Type] OR "controlled clinical trial"[Publication Type] OR "government publication"[Publication Type] OR "historical article"[Publication Type] OR "introductory journal article"[Publication Type] OR "journal article"[Publication Type] OR "multicenter study"[Publication Type] OR "observational study"[Publication Type] OR "overall"[Publication Type] OR "pragmatic clinical trial"[Publication Type] OR "randomized controlled trial"[Publication Type] OR "technical report"[Publication Type] OR "twin study"[Publication Type] OR "validation study"[Publication Type]) AND "humans"[MeSH Terms] AND 1950/01/01:2020/12/31[Date - Publication] AND ("adult"[MeSH Terms] OR "young adult"[MeSH Terms] OR "adult"[MeSH Terms:noexp] OR ("middle aged"[MeSH Terms] OR "aged"[MeSH Terms]) OR "middle aged"[MeSH Terms] OR "aged"[MeSH Terms] OR "aged, 80 and over"[MeSH Terms])) AND (("saudi arabia"[MeSH Terms] OR ("saudi"[All Fields] AND "arabia"[All Fields]) OR "saudi arabia"[All Fields] OR "Gulf"[All Fields] OR "KSA"[All Fields]) AND ("hasabstract"[All Fields] AND ("pubmed books"[Filter] OR "case reports"[Publication Type] OR "clinical study"[Publication Type] OR "clinical trial"[Publication Type] OR "clinical trial, phase i"[Publication Type] OR "clinical trial, phase ii"[Publication Type] OR "clinical trial, phase iii"[Publication Type] OR "clinical trial, phase iv"[Publication Type] OR "comparative study"[Publication Type] OR "controlled clinical trial"[Publication Type] OR "government publication"[Publication Type] OR "historical article"[Publication Type] OR "introductory journal article"[Publication Type] OR "journal article"[Publication Type] OR "multicenter study"[Publication Type] OR "observational study"[Publication Type] OR "overall"[Publication Type] OR "pragmatic clinical trial"[Publication Type] OR "randomized controlled trial"[Publication Type] OR "technical report"[Publication Type] OR "twin study"[Publication Type] OR "validation study"[Publication Type]) AND "humans"[MeSH Terms] AND 1950/01/01:2020/12/31[Date - Publication] AND ("adult"[MeSH Terms] OR "young adult"[MeSH Terms] OR "adult"[MeSH Terms:noexp] OR ("middle aged"[MeSH Terms] OR "aged"[MeSH Terms]) OR "middle aged"[MeSH Terms] OR "aged"[MeSH Terms] OR "aged, 80 and over"[MeSH Terms]))) AND ("hasabstract"[All Fields] AND ("pubmed books"[Filter] OR "case reports"[Publication Type] OR "clinical study"[Publication Type] OR "clinical trial"[Publication Type] OR "clinical trial, phase i"[Publication Type] OR "clinical trial, phase ii"[Publication Type] OR "clinical trial, phase iii"[Publication Type] OR "clinical trial, phase iv"[Publication Type] OR "comparative study"[Publication Type] OR "controlled clinical trial"[Publication Type] OR "government publication"[Publication Type] OR "historical article"[Publication Type] OR "introductory journal article"[Publication Type] OR "journal article"[Publication Type] OR "multicenter study"[Publication Type] OR "observational study"[Publication Type] OR "overall"[Publication Type] OR "pragmatic clinical trial"[Publication Type] OR "randomized controlled trial"[Publication Type] OR "technical report"[Publication Type] OR "twin study"[Publication Type] OR "validation study"[Publication Type]) AND "humans"[MeSH Terms] AND 1950/01/01:2020/12/31[Date - Publication] AND ("adult"[MeSH Terms] OR "young adult"[MeSH Terms] OR "adult"[MeSH Terms:noexp] OR ("middle aged"[MeSH Terms] OR "aged"[MeSH Terms]) OR "middle aged"[MeSH Terms] OR "aged"[MeSH Terms] OR "aged, 80 and over"[MeSH Terms])) AND (("risk*"[All Fields] OR "epidem*"[All Fields] OR "behav*"[All Fields] OR "habit*"[All Fields] OR ("life style"[MeSH Terms] OR ("life"[All Fields] AND "style"[All Fields]) OR "life style"[All Fields] OR "lifestyle"[All Fields] OR "lifestyles"[All Fields]) OR ("epidemiology"[MeSH Subheading] OR "epidemiology"[All Fields] OR "prevalence"[All Fields] OR "prevalence"[MeSH Terms] OR "prevalance"[All Fields] OR "prevalences"[All Fields] OR "prevalence s"[All Fields] OR "prevalent"[All Fields] OR "prevalently"[All Fields] OR "prevalents"[All Fields])) AND ("hasabstract"[All Fields] AND ("pubmed books"[Filter] OR "case reports"[Publication Type] OR "clinical study"[Publication Type] OR "clinical trial"[Publication Type] OR "clinical trial, phase i"[Publication Type] OR "clinical trial, phase ii"[Publication Type] OR "clinical trial, phase iii"[Publication Type] OR "clinical trial, phase iv"[Publication Type] OR "comparative study"[Publication Type] OR "controlled clinical trial"[Publication Type] OR "government publication"[Publication Type] OR "historical article"[Publication Type] OR "introductory journal article"[Publication Type] OR "journal article"[Publication Type] OR "multicenter study"[Publication Type] OR "observational study"[Publication Type] OR "overall"[Publication Type] OR "pragmatic clinical trial"[Publication Type] OR "randomized controlled trial"[Publication Type] OR "technical report"[Publication Type] OR "twin study"[Publication Type] OR "validation study"[Publication Type]) AND "humans"[MeSH Terms] AND 1950/01/01:2020/12/31[Date - Publication] AND ("adult"[MeSH Terms] OR "young adult"[MeSH Terms] OR "adult"[MeSH Terms:noexp] OR ("middle aged"[MeSH Terms] OR "aged"[MeSH Terms]) OR "middle aged"[MeSH Terms] OR "aged"[MeSH Terms] OR "aged, 80 and over"[MeSH Terms]))) 6. "gene*"[All Fields] OR ("polymorphic"[All Fields] OR "polymorphics"[All Fields] OR "polymorphism s"[All Fields] OR "polymorphism, genetic"[MeSH Terms] OR ("polymorphism"[All Fields] AND "genetic"[All Fields]) OR "genetic polymorphism"[All Fields] OR "polymorphism"[All Fields] OR "polymorphisms"[All Fields]) OR ("phenotype"[MeSH Terms] OR "phenotype"[All Fields] OR "phenotypes"[All Fields] OR "phenotyped"[All Fields] OR "phenotypic"[All Fields] OR "phenotypical"[All Fields] OR "phenotypically"[All Fields] OR "phenotyping"[All Fields] OR "phenotypings"[All Fields]) OR ("genome"[MeSH Terms] OR "genome"[All Fields] OR "genomes"[All Fields] OR "genome s"[All Fields] OR "genomically"[All Fields] OR "genomics"[MeSH Terms] OR "genomics"[All Fields] OR "genomic"[All Fields]) 7. ("obeses"[All Fields] OR "obesity"[MeSH Terms] OR "obesity"[All Fields] OR "obese"[All Fields] OR "obesities"[All Fields] OR "obesity s"[All Fields] OR ("overweight"[MeSH Terms] OR "overweight"[All Fields] OR "overweighted"[All Fields] OR "overweightness"[All Fields] OR "overweights"[All Fields]) OR ("metabolic syndrome"[MeSH Terms] OR ("metabolic"[All Fields] AND "syndrome"[All Fields]) OR "metabolic syndrome"[All Fields]) OR "waist-circumference"[All Fields] OR "BMI"[All Fields]) AND ("hasabstract"[All Fields] AND ("pubmed books"[Filter] OR "case reports"[Publication Type] OR "clinical study"[Publication Type] OR "clinical trial"[Publication Type] OR "clinical trial, phase i"[Publication Type] OR "clinical trial, phase ii"[Publication Type] OR "clinical trial, phase iii"[Publication Type] OR "clinical trial, phase iv"[Publication Type] OR "comparative study"[Publication Type] OR "controlled clinical trial"[Publication Type] OR "government publication"[Publication Type] OR "historical article"[Publication Type] OR "introductory journal article"[Publication Type] OR "journal article"[Publication Type] OR "multicenter study"[Publication Type] OR "observational study"[Publication Type] OR "overall"[Publication Type] OR "pragmatic clinical trial"[Publication Type] OR "randomized controlled trial"[Publication Type] OR "technical report"[Publication Type] OR "twin study"[Publication Type] OR "validation study"[Publication Type]) AND "humans"[MeSH Terms] AND 1950/01/01:2020/12/31[Date - Publication] AND ("adult"[MeSH Terms] OR "young adult"[MeSH Terms] OR "adult"[MeSH Terms:noexp] OR ("middle aged"[MeSH Terms] OR "aged"[MeSH Terms]) OR "middle aged"[MeSH Terms] OR "aged"[MeSH Terms] OR "aged, 80 and over"[MeSH Terms])) AND (("saudi arabia"[MeSH Terms] OR ("saudi"[All Fields] AND "arabia"[All Fields]) OR "saudi arabia"[All Fields] OR "Gulf"[All Fields] OR "KSA"[All Fields]) AND ("hasabstract"[All Fields] AND ("pubmed books"[Filter] OR "case reports"[Publication Type] OR "clinical study"[Publication Type] OR "clinical trial"[Publication Type] OR "clinical trial, phase i"[Publication Type] OR "clinical trial, phase ii"[Publication Type] OR "clinical trial, phase iii"[Publication Type] OR "clinical trial, phase iv"[Publication Type] OR "comparative study"[Publication Type] OR "controlled clinical trial"[Publication Type] OR "government publication"[Publication Type] OR "historical article"[Publication Type] OR "introductory journal article"[Publication Type] OR "journal article"[Publication Type] OR "multicenter study"[Publication Type] OR "observational study"[Publication Type] OR "overall"[Publication Type] OR "pragmatic clinical trial"[Publication Type] OR "randomized controlled trial"[Publication Type] OR "technical report"[Publication Type] OR "twin study"[Publication Type] OR "validation study"[Publication Type]) AND "humans"[MeSH Terms] AND 1950/01/01:2020/12/31[Date - Publication] AND ("adult"[MeSH Terms] OR "young adult"[MeSH Terms] OR "adult"[MeSH Terms:noexp] OR ("middle aged"[MeSH Terms] OR "aged"[MeSH Terms]) OR "middle aged"[MeSH Terms] OR "aged"[MeSH Terms] OR "aged, 80 and over"[MeSH Terms]))) AND ("hasabstract"[All Fields] AND ("pubmed books"[Filter] OR "case reports"[Publication Type] OR "clinical study"[Publication Type] OR "clinical trial"[Publication Type] OR "clinical trial, phase i"[Publication Type] OR "clinical trial, phase ii"[Publication Type] OR "clinical trial, phase iii"[Publication Type] OR "clinical trial, phase iv"[Publication Type] OR "comparative study"[Publication Type] OR "controlled clinical trial"[Publication Type] OR "government publication"[Publication Type] OR "historical article"[Publication Type] OR "introductory journal article"[Publication Type] OR "journal article"[Publication Type] OR "multicenter study"[Publication Type] OR "observational study"[Publication Type] OR "overall"[Publication Type] OR "pragmatic clinical trial"[Publication Type] OR "randomized controlled trial"[Publication Type] OR "technical report"[Publication Type] OR "twin study"[Publication Type] OR "validation study"[Publication Type]) AND "humans"[MeSH Terms] AND 1950/01/01:2020/12/31[Date - Publication] AND ("adult"[MeSH Terms] OR "young adult"[MeSH Terms] OR "adult"[MeSH Terms:noexp] OR ("middle aged"[MeSH Terms] OR "aged"[MeSH Terms]) OR "middle aged"[MeSH Terms] OR "aged"[MeSH Terms] OR "aged, 80 and over"[MeSH Terms])) AND (("gene*"[All Fields] OR ("polymorphic"[All Fields] OR "polymorphics"[All Fields] OR "polymorphism s"[All Fields] OR "polymorphism, genetic"[MeSH Terms] OR ("polymorphism"[All Fields] AND "genetic"[All Fields]) OR "genetic polymorphism"[All Fields] OR "polymorphism"[All Fields] OR "polymorphisms"[All Fields]) OR ("phenotype"[MeSH Terms] OR "phenotype"[All Fields] OR "phenotypes"[All Fields] OR "phenotyped"[All Fields] OR "phenotypic"[All Fields] OR "phenotypical"[All Fields] OR "phenotypically"[All Fields] OR "phenotyping"[All Fields] OR "phenotypings"[All Fields]) OR ("genome"[MeSH Terms] OR "genome"[All Fields] OR "genomes"[All Fields] OR "genome s"[All Fields] OR "genomically"[All Fields] OR "genomics"[MeSH Terms] OR "genomics"[All Fields] OR "genomic"[All Fields])) AND ("hasabstract"[All Fields] AND ("pubmed books"[Filter] OR "case reports"[Publication Type] OR "clinical study"[Publication Type] OR "clinical trial"[Publication Type] OR "clinical trial, phase i"[Publication Type] OR "clinical trial, phase ii"[Publication Type] OR "clinical trial, phase iii"[Publication Type] OR "clinical trial, phase iv"[Publication Type] OR "comparative study"[Publication Type] OR "controlled clinical trial"[Publication Type] OR "government publication"[Publication Type] OR "historical article"[Publication Type] OR "introductory journal article"[Publication Type] OR "journal article"[Publication Type] OR "multicenter study"[Publication Type] OR "observational study"[Publication Type] OR "overall"[Publication Type] OR "pragmatic clinical trial"[Publication Type] OR "randomized controlled trial"[Publication Type] OR "technical report"[Publication Type] OR "twin study"[Publication Type] OR "validation study"[Publication Type]) AND "humans"[MeSH Terms] AND 1950/01/01:2020/12/31[Date - Publication] AND ("adult"[MeSH Terms] OR "young adult"[MeSH Terms] OR "adult"[MeSH Terms:noexp] OR ("middle aged"[MeSH Terms] OR "aged"[MeSH Terms]) OR "middle aged"[MeSH Terms] OR "aged"[MeSH Terms] OR "aged, 80 and over"[MeSH Terms]))) 8. "exercise"[MeSH Terms] OR "exercise"[All Fields] OR "exercises"[All Fields] OR "exercise therapy"[MeSH Terms] OR ("exercise"[All Fields] AND "therapy"[All Fields]) OR "exercise therapy"[All Fields] OR "exercise s"[All Fields] OR "exercised"[All Fields] OR "exerciser"[All Fields] OR "exercisers"[All Fields] OR "exercising"[All Fields] OR ("exercise"[MeSH Terms] OR "exercise"[All Fields] OR ("physical"[All Fields] AND "activity"[All Fields]) OR "physical activity"[All Fields]) OR ("sedentaries"[All Fields] OR "sedentariness"[All Fields] OR "sedentary"[All Fields]) 9. ("obeses"[All Fields] OR "obesity"[MeSH Terms] OR "obesity"[All Fields] OR "obese"[All Fields] OR "obesities"[All Fields] OR "obesity s"[All Fields] OR ("overweight"[MeSH Terms] OR "overweight"[All Fields] OR "overweighted"[All Fields] OR "overweightness"[All Fields] OR "overweights"[All Fields]) OR ("metabolic syndrome"[MeSH Terms] OR ("metabolic"[All Fields] AND "syndrome"[All Fields]) OR "metabolic syndrome"[All Fields]) OR "waist-circumference"[All Fields] OR "BMI"[All Fields]) AND ("hasabstract"[All Fields] AND ("pubmed books"[Filter] OR "case reports"[Publication Type] OR "clinical study"[Publication Type] OR "clinical trial"[Publication Type] OR "clinical trial, phase i"[Publication Type] OR "clinical trial, phase ii"[Publication Type] OR "clinical trial, phase iii"[Publication Type] OR "clinical trial, phase iv"[Publication Type] OR "comparative study"[Publication Type] OR "controlled clinical trial"[Publication Type] OR "government publication"[Publication Type] OR "historical article"[Publication Type] OR "introductory journal article"[Publication Type] OR "journal article"[Publication Type] OR "multicenter study"[Publication Type] OR "observational study"[Publication Type] OR "overall"[Publication Type] OR "pragmatic clinical trial"[Publication Type] OR "randomized controlled trial"[Publication Type] OR "technical report"[Publication Type] OR "twin study"[Publication Type] OR "validation study"[Publication Type]) AND "humans"[MeSH Terms] AND 1950/01/01:2020/12/31[Date - Publication] AND ("adult"[MeSH Terms] OR "young adult"[MeSH Terms] OR "adult"[MeSH Terms:noexp] OR ("middle aged"[MeSH Terms] OR "aged"[MeSH Terms]) OR "middle aged"[MeSH Terms] OR "aged"[MeSH Terms] OR "aged, 80 and over"[MeSH Terms])) AND (("saudi arabia"[MeSH Terms] OR ("saudi"[All Fields] AND "arabia"[All Fields]) OR "saudi arabia"[All Fields] OR "Gulf"[All Fields] OR "KSA"[All Fields]) AND ("hasabstract"[All Fields] AND ("pubmed books"[Filter] OR "case reports"[Publication Type] OR "clinical study"[Publication Type] OR "clinical trial"[Publication Type] OR "clinical trial, phase i"[Publication Type] OR "clinical trial, phase ii"[Publication Type] OR "clinical trial, phase iii"[Publication Type] OR "clinical trial, phase iv"[Publication Type] OR "comparative study"[Publication Type] OR "controlled clinical trial"[Publication Type] OR "government publication"[Publication Type] OR "historical article"[Publication Type] OR "introductory journal article"[Publication Type] OR "journal article"[Publication Type] OR "multicenter study"[Publication Type] OR "observational study"[Publication Type] OR "overall"[Publication Type] OR "pragmatic clinical trial"[Publication Type] OR "randomized controlled trial"[Publication Type] OR "technical report"[Publication Type] OR "twin study"[Publication Type] OR "validation study"[Publication Type]) AND "humans"[MeSH Terms] AND 1950/01/01:2020/12/31[Date - Publication] AND ("adult"[MeSH Terms] OR "young adult"[MeSH Terms] OR "adult"[MeSH Terms:noexp] OR ("middle aged"[MeSH Terms] OR "aged"[MeSH Terms]) OR "middle aged"[MeSH Terms] OR "aged"[MeSH Terms] OR "aged, 80 and over"[MeSH Terms]))) AND ("hasabstract"[All Fields] AND ("pubmed books"[Filter] OR "case reports"[Publication Type] OR "clinical study"[Publication Type] OR "clinical trial"[Publication Type] OR "clinical trial, phase i"[Publication Type] OR "clinical trial, phase ii"[Publication Type] OR "clinical trial, phase iii"[Publication Type] OR "clinical trial, phase iv"[Publication Type] OR "comparative study"[Publication Type] OR "controlled clinical trial"[Publication Type] OR "government publication"[Publication Type] OR "historical article"[Publication Type] OR "introductory journal article"[Publication Type] OR "journal article"[Publication Type] OR "multicenter study"[Publication Type] OR "observational study"[Publication Type] OR "overall"[Publication Type] OR "pragmatic clinical trial"[Publication Type] OR "randomized controlled trial"[Publication Type] OR "technical report"[Publication Type] OR "twin study"[Publication Type] OR "validation study"[Publication Type]) AND "humans"[MeSH Terms] AND 1950/01/01:2020/12/31[Date - Publication] AND ("adult"[MeSH Terms] OR "young adult"[MeSH Terms] OR "adult"[MeSH Terms:noexp] OR ("middle aged"[MeSH Terms] OR "aged"[MeSH Terms]) OR "middle aged"[MeSH Terms] OR "aged"[MeSH Terms] OR "aged, 80 and over"[MeSH Terms])) AND (("exercise"[MeSH Terms] OR "exercise"[All Fields] OR "exercises"[All Fields] OR "exercise therapy"[MeSH Terms] OR ("exercise"[All Fields] AND "therapy"[All Fields]) OR "exercise therapy"[All Fields] OR "exercise s"[All Fields] OR "exercised"[All Fields] OR "exerciser"[All Fields] OR "exercisers"[All Fields] OR "exercising"[All Fields] OR ("exercise"[MeSH Terms] OR "exercise"[All Fields] OR ("physical"[All Fields] AND "activity"[All Fields]) OR "physical activity"[All Fields]) OR ("sedentaries"[All Fields] OR "sedentariness"[All Fields] OR "sedentary"[All Fields])) AND ("hasabstract"[All Fields] AND ("pubmed books"[Filter] OR "case reports"[Publication Type] OR "clinical study"[Publication Type] OR "clinical trial"[Publication Type] OR "clinical trial, phase i"[Publication Type] OR "clinical trial, phase ii"[Publication Type] OR "clinical trial, phase iii"[Publication Type] OR "clinical trial, phase iv"[Publication Type] OR "comparative study"[Publication Type] OR "controlled clinical trial"[Publication Type] OR "government publication"[Publication Type] OR "historical article"[Publication Type] OR "introductory journal article"[Publication Type] OR "journal article"[Publication Type] OR "multicenter study"[Publication Type] OR "observational study"[Publication Type] OR "overall"[Publication Type] OR "pragmatic clinical trial"[Publication Type] OR "randomized controlled trial"[Publication Type] OR "technical report"[Publication Type] OR "twin study"[Publication Type] OR "validation study"[Publication Type]) AND "humans"[MeSH Terms] AND 1950/01/01:2020/12/31[Date - Publication] AND ("adult"[MeSH Terms] OR "young adult"[MeSH Terms] OR "adult"[MeSH Terms:noexp] OR ("middle aged"[MeSH Terms] OR "aged"[MeSH Terms]) OR "middle aged"[MeSH Terms] OR "aged"[MeSH Terms] OR "aged, 80 and over"[MeSH Terms]))) 10. "food"[MeSH Terms] OR "food"[All Fields] OR "diet*"[All Fields] OR "eating"[MeSH Terms] OR "eating"[All Fields] OR "feeding"[All Fields] OR "feedings"[All Fields] OR "feeds"[All Fields] 11. ("obeses"[All Fields] OR "obesity"[MeSH Terms] OR "obesity"[All Fields] OR "obese"[All Fields] OR "obesities"[All Fields] OR "obesity s"[All Fields] OR ("overweight"[MeSH Terms] OR "overweight"[All Fields] OR "overweighted"[All Fields] OR "overweightness"[All Fields] OR "overweights"[All Fields]) OR ("metabolic syndrome"[MeSH Terms] OR ("metabolic"[All Fields] AND "syndrome"[All Fields]) OR "metabolic syndrome"[All Fields]) OR "waist-circumference"[All Fields] OR "BMI"[All Fields]) AND ("hasabstract"[All Fields] AND ("pubmed books"[Filter] OR "case reports"[Publication Type] OR "clinical study"[Publication Type] OR "clinical trial"[Publication Type] OR "clinical trial, phase i"[Publication Type] OR "clinical trial, phase ii"[Publication Type] OR "clinical trial, phase iii"[Publication Type] OR "clinical trial, phase iv"[Publication Type] OR "comparative study"[Publication Type] OR "controlled clinical trial"[Publication Type] OR "government publication"[Publication Type] OR "historical article"[Publication Type] OR "introductory journal article"[Publication Type] OR "journal article"[Publication Type] OR "multicenter study"[Publication Type] OR "observational study"[Publication Type] OR "overall"[Publication Type] OR "pragmatic clinical trial"[Publication Type] OR "randomized controlled trial"[Publication Type] OR "technical report"[Publication Type] OR "twin study"[Publication Type] OR "validation study"[Publication Type]) AND "humans"[MeSH Terms] AND 1950/01/01:2020/12/31[Date - Publication] AND ("adult"[MeSH Terms] OR "young adult"[MeSH Terms] OR "adult"[MeSH Terms:noexp] OR ("middle aged"[MeSH Terms] OR "aged"[MeSH Terms]) OR "middle aged"[MeSH Terms] OR "aged"[MeSH Terms] OR "aged, 80 and over"[MeSH Terms])) AND (("saudi arabia"[MeSH Terms] OR ("saudi"[All Fields] AND "arabia"[All Fields]) OR "saudi arabia"[All Fields] OR "Gulf"[All Fields] OR "KSA"[All Fields]) AND ("hasabstract"[All Fields] AND ("pubmed books"[Filter] OR "case reports"[Publication Type] OR "clinical study"[Publication Type] OR "clinical trial"[Publication Type] OR "clinical trial, phase i"[Publication Type] OR "clinical trial, phase ii"[Publication Type] OR "clinical trial, phase iii"[Publication Type] OR "clinical trial, phase iv"[Publication Type] OR "comparative study"[Publication Type] OR "controlled clinical trial"[Publication Type] OR "government publication"[Publication Type] OR "historical article"[Publication Type] OR "introductory journal article"[Publication Type] OR "journal article"[Publication Type] OR "multicenter study"[Publication Type] OR "observational study"[Publication Type] OR "overall"[Publication Type] OR "pragmatic clinical trial"[Publication Type] OR "randomized controlled trial"[Publication Type] OR "technical report"[Publication Type] OR "twin study"[Publication Type] OR "validation study"[Publication Type]) AND "humans"[MeSH Terms] AND 1950/01/01:2020/12/31[Date - Publication] AND ("adult"[MeSH Terms] OR "young adult"[MeSH Terms] OR "adult"[MeSH Terms:noexp] OR ("middle aged"[MeSH Terms] OR "aged"[MeSH Terms]) OR "middle aged"[MeSH Terms] OR "aged"[MeSH Terms] OR "aged, 80 and over"[MeSH Terms]))) AND ("hasabstract"[All Fields] AND ("pubmed books"[Filter] OR "case reports"[Publication Type] OR "clinical study"[Publication Type] OR "clinical trial"[Publication Type] OR "clinical trial, phase i"[Publication Type] OR "clinical trial, phase ii"[Publication Type] OR "clinical trial, phase iii"[Publication Type] OR "clinical trial, phase iv"[Publication Type] OR "comparative study"[Publication Type] OR "controlled clinical trial"[Publication Type] OR "government publication"[Publication Type] OR "historical article"[Publication Type] OR "introductory journal article"[Publication Type] OR "journal article"[Publication Type] OR "multicenter study"[Publication Type] OR "observational study"[Publication Type] OR "overall"[Publication Type] OR "pragmatic clinical trial"[Publication Type] OR "randomized controlled trial"[Publication Type] OR "technical report"[Publication Type] OR "twin study"[Publication Type] OR "validation study"[Publication Type]) AND "humans"[MeSH Terms] AND 1950/01/01:2020/12/31[Date - Publication] AND ("adult"[MeSH Terms] OR "young adult"[MeSH Terms] OR "adult"[MeSH Terms:noexp] OR ("middle aged"[MeSH Terms] OR "aged"[MeSH Terms]) OR "middle aged"[MeSH Terms] OR "aged"[MeSH Terms] OR "aged, 80 and over"[MeSH Terms])) AND (("food"[MeSH Terms] OR "food"[All Fields] OR "diet*"[All Fields] OR ("eating"[MeSH Terms] OR "eating"[All Fields]) OR ("feeding"[All Fields] OR "feedings"[All Fields] OR "feeds"[All Fields])) AND ("hasabstract"[All Fields] AND ("pubmed books"[Filter] OR "case reports"[Publication Type] OR "clinical study"[Publication Type] OR "clinical trial"[Publication Type] OR "clinical trial, phase i"[Publication Type] OR "clinical trial, phase ii"[Publication Type] OR "clinical trial, phase iii"[Publication Type] OR "clinical trial, phase iv"[Publication Type] OR "comparative study"[Publication Type] OR "controlled clinical trial"[Publication Type] OR "government publication"[Publication Type] OR "historical article"[Publication Type] OR "introductory journal article"[Publication Type] OR "journal article"[Publication Type] OR "multicenter study"[Publication Type] OR "observational study"[Publication Type] OR "overall"[Publication Type] OR "pragmatic clinical trial"[Publication Type] OR "randomized controlled trial"[Publication Type] OR "technical report"[Publication Type] OR "twin study"[Publication Type] OR "validation study"[Publication Type]) AND "humans"[MeSH Terms] AND 1950/01/01:2020/12/31[Date - Publication] AND ("adult"[MeSH Terms] OR "young adult"[MeSH Terms] OR "adult"[MeSH Terms:noexp] OR ("middle aged"[MeSH Terms] OR "aged"[MeSH Terms]) OR "middle aged"[MeSH Terms] OR "aged"[MeSH Terms] OR "aged, 80 and over"[MeSH Terms]))) |
| B) Research theme 2: How are effective treatments for obesity currently being delivered to adults with obesity in SA? |
| 1. "obeses"[All Fields] OR "obesity"[MeSH Terms] OR "obesity"[All Fields] OR "obese"[All Fields] OR "obesities"[All Fields] OR "obesity s"[All Fields] OR ("overweight"[MeSH Terms] OR "overweight"[All Fields] OR "overweighted"[All Fields] OR "overweightness"[All Fields] OR "overweights"[All Fields]) OR ("metabolic syndrome"[MeSH Terms] OR ("metabolic"[All Fields] AND "syndrome"[All Fields]) OR "metabolic syndrome"[All Fields]) OR "waist-circumference"[All Fields] OR "BMI"[All Fields] 2. "saudi arabia"[MeSH Terms] OR ("saudi"[All Fields] AND "arabia"[All Fields]) OR "saudi arabia"[All Fields] OR "Gulf"[All Fields] OR "KSA"[All Fields] 3. ("obeses"[All Fields] OR "obesity"[MeSH Terms] OR "obesity"[All Fields] OR "obese"[All Fields] OR "obesities"[All Fields] OR "obesity s"[All Fields] OR ("overweight"[MeSH Terms] OR "overweight"[All Fields] OR "overweighted"[All Fields] OR "overweightness"[All Fields] OR "overweights"[All Fields]) OR ("metabolic syndrome"[MeSH Terms] OR ("metabolic"[All Fields] AND "syndrome"[All Fields]) OR "metabolic syndrome"[All Fields]) OR "waist-circumference"[All Fields] OR "BMI"[All Fields]) AND ("hasabstract"[All Fields] AND ("case reports"[Publication Type] OR "clinical study"[Publication Type] OR "clinical trial"[Publication Type] OR "clinical trial, phase i"[Publication Type] OR "clinical trial, phase ii"[Publication Type] OR "clinical trial, phase iii"[Publication Type] OR "clinical trial, phase iv"[Publication Type] OR "comparative study"[Publication Type] OR "controlled clinical trial"[Publication Type] OR "government publication"[Publication Type] OR "historical article"[Publication Type] OR "introductory journal article"[Publication Type] OR "journal article"[Publication Type] OR "multicenter study"[Publication Type] OR "observational study"[Publication Type] OR "overall"[Publication Type] OR "pragmatic clinical trial"[Publication Type] OR "randomized controlled trial"[Publication Type] OR "twin study"[Publication Type] OR "validation study"[Publication Type]) AND "humans"[MeSH Terms] AND 1950/01/01:2020/12/31[Date - Publication] AND ("adult"[MeSH Terms] OR "young adult"[MeSH Terms] OR "adult"[MeSH Terms:noexp] OR ("middle aged"[MeSH Terms] OR "aged"[MeSH Terms]) OR "middle aged"[MeSH Terms] OR "aged"[MeSH Terms] OR "aged, 80 and over"[MeSH Terms])) AND (("saudi arabia"[MeSH Terms] OR ("saudi"[All Fields] AND "arabia"[All Fields]) OR "saudi arabia"[All Fields] OR "Gulf"[All Fields] OR "KSA"[All Fields]) AND ("hasabstract"[All Fields] AND ("case reports"[Publication Type] OR "clinical study"[Publication Type] OR "clinical trial"[Publication Type] OR "clinical trial, phase i"[Publication Type] OR "clinical trial, phase ii"[Publication Type] OR "clinical trial, phase iii"[Publication Type] OR "clinical trial, phase iv"[Publication Type] OR "comparative study"[Publication Type] OR "controlled clinical trial"[Publication Type] OR "government publication"[Publication Type] OR "historical article"[Publication Type] OR "introductory journal article"[Publication Type] OR "journal article"[Publication Type] OR "multicenter study"[Publication Type] OR "observational study"[Publication Type] OR "overall"[Publication Type] OR "pragmatic clinical trial"[Publication Type] OR "randomized controlled trial"[Publication Type] OR "twin study"[Publication Type] OR "validation study"[Publication Type]) AND "humans"[MeSH Terms] AND 1950/01/01:2020/12/31[Date - Publication] AND ("adult"[MeSH Terms] OR "young adult"[MeSH Terms] OR "adult"[MeSH Terms:noexp] OR ("middle aged"[MeSH Terms] OR "aged"[MeSH Terms]) OR "middle aged"[MeSH Terms] OR "aged"[MeSH Terms] OR "aged, 80 and over"[MeSH Terms]))) 4. ("intervention*"[All Fields] OR "weight management"[All Fields] OR "program*"[All Fields] OR "behav*"[All Fields] OR ("life style"[MeSH Terms] OR ("life"[All Fields] AND "style"[All Fields]) OR "life style"[All Fields] OR "lifestyle"[All Fields] OR "lifestyles"[All Fields]) OR "weight-control"[All Fields] OR ("therapeutics"[MeSH Terms] OR "therapeutics"[All Fields] OR "treatments"[All Fields] OR "therapy"[MeSH Subheading] OR "therapy"[All Fields] OR "treatment"[All Fields] OR "treatment s"[All Fields])) AND ("hasabstract"[All Fields] AND ("case reports"[Publication Type] OR "clinical study"[Publication Type] OR "clinical trial"[Publication Type] OR "clinical trial, phase i"[Publication Type] OR "clinical trial, phase ii"[Publication Type] OR "clinical trial, phase iii"[Publication Type] OR "clinical trial, phase iv"[Publication Type] OR "comparative study"[Publication Type] OR "controlled clinical trial"[Publication Type] OR "government publication"[Publication Type] OR "historical article"[Publication Type] OR "introductory journal article"[Publication Type] OR "journal article"[Publication Type] OR "multicenter study"[Publication Type] OR "observational study"[Publication Type] OR "overall"[Publication Type] OR "pragmatic clinical trial"[Publication Type] OR "randomized controlled trial"[Publication Type] OR "twin study"[Publication Type] OR "validation study"[Publication Type]) AND "humans"[MeSH Terms] AND 1950/01/01:2020/12/31[Date - Publication] AND ("adult"[MeSH Terms] OR "young adult"[MeSH Terms] OR "adult"[MeSH Terms:noexp] OR ("middle aged"[MeSH Terms] OR "aged"[MeSH Terms]) OR "middle aged"[MeSH Terms] OR "aged"[MeSH Terms] OR "aged, 80 and over"[MeSH Terms])) 5. ("obeses"[All Fields] OR "obesity"[MeSH Terms] OR "obesity"[All Fields] OR "obese"[All Fields] OR "obesities"[All Fields] OR "obesity s"[All Fields] OR ("overweight"[MeSH Terms] OR "overweight"[All Fields] OR "overweighted"[All Fields] OR "overweightness"[All Fields] OR "overweights"[All Fields]) OR ("metabolic syndrome"[MeSH Terms] OR ("metabolic"[All Fields] AND "syndrome"[All Fields]) OR "metabolic syndrome"[All Fields]) OR "waist-circumference"[All Fields] OR "BMI"[All Fields]) AND ("hasabstract"[All Fields] AND ("case reports"[Publication Type] OR "clinical study"[Publication Type] OR "clinical trial"[Publication Type] OR "clinical trial, phase i"[Publication Type] OR "clinical trial, phase ii"[Publication Type] OR "clinical trial, phase iii"[Publication Type] OR "clinical trial, phase iv"[Publication Type] OR "comparative study"[Publication Type] OR "controlled clinical trial"[Publication Type] OR "government publication"[Publication Type] OR "historical article"[Publication Type] OR "introductory journal article"[Publication Type] OR "journal article"[Publication Type] OR "multicenter study"[Publication Type] OR "observational study"[Publication Type] OR "overall"[Publication Type] OR "pragmatic clinical trial"[Publication Type] OR "randomized controlled trial"[Publication Type] OR "twin study"[Publication Type] OR "validation study"[Publication Type]) AND "humans"[MeSH Terms] AND 1950/01/01:2020/12/31[Date - Publication] AND ("adult"[MeSH Terms] OR "young adult"[MeSH Terms] OR "adult"[MeSH Terms:noexp] OR ("middle aged"[MeSH Terms] OR "aged"[MeSH Terms]) OR "middle aged"[MeSH Terms] OR "aged"[MeSH Terms] OR "aged, 80 and over"[MeSH Terms])) AND (("saudi arabia"[MeSH Terms] OR ("saudi"[All Fields] AND "arabia"[All Fields]) OR "saudi arabia"[All Fields] OR "Gulf"[All Fields] OR "KSA"[All Fields]) AND ("hasabstract"[All Fields] AND ("case reports"[Publication Type] OR "clinical study"[Publication Type] OR "clinical trial"[Publication Type] OR "clinical trial, phase i"[Publication Type] OR "clinical trial, phase ii"[Publication Type] OR "clinical trial, phase iii"[Publication Type] OR "clinical trial, phase iv"[Publication Type] OR "comparative study"[Publication Type] OR "controlled clinical trial"[Publication Type] OR "government publication"[Publication Type] OR "historical article"[Publication Type] OR "introductory journal article"[Publication Type] OR "journal article"[Publication Type] OR "multicenter study"[Publication Type] OR "observational study"[Publication Type] OR "overall"[Publication Type] OR "pragmatic clinical trial"[Publication Type] OR "randomized controlled trial"[Publication Type] OR "twin study"[Publication Type] OR "validation study"[Publication Type]) AND "humans"[MeSH Terms] AND 1950/01/01:2020/12/31[Date - Publication] AND ("adult"[MeSH Terms] OR "young adult"[MeSH Terms] OR "adult"[MeSH Terms:noexp] OR ("middle aged"[MeSH Terms] OR "aged"[MeSH Terms]) OR "middle aged"[MeSH Terms] OR "aged"[MeSH Terms] OR "aged, 80 and over"[MeSH Terms]))) AND ("hasabstract"[All Fields] AND ("case reports"[Publication Type] OR "clinical study"[Publication Type] OR "clinical trial"[Publication Type] OR "clinical trial, phase i"[Publication Type] OR "clinical trial, phase ii"[Publication Type] OR "clinical trial, phase iii"[Publication Type] OR "clinical trial, phase iv"[Publication Type] OR "comparative study"[Publication Type] OR "controlled clinical trial"[Publication Type] OR "government publication"[Publication Type] OR "historical article"[Publication Type] OR "introductory journal article"[Publication Type] OR "journal article"[Publication Type] OR "multicenter study"[Publication Type] OR "observational study"[Publication Type] OR "overall"[Publication Type] OR "pragmatic clinical trial"[Publication Type] OR "randomized controlled trial"[Publication Type] OR "twin study"[Publication Type] OR "validation study"[Publication Type]) AND "humans"[MeSH Terms] AND 1950/01/01:2020/12/31[Date - Publication] AND ("adult"[MeSH Terms] OR "young adult"[MeSH Terms] OR "adult"[MeSH Terms:noexp] OR ("middle aged"[MeSH Terms] OR "aged"[MeSH Terms]) OR "middle aged"[MeSH Terms] OR "aged"[MeSH Terms] OR "aged, 80 and over"[MeSH Terms])) AND (("intervention*"[All Fields] OR "weight management"[All Fields] OR "program*"[All Fields] OR "behav*"[All Fields] OR ("life style"[MeSH Terms] OR ("life"[All Fields] AND "style"[All Fields]) OR "life style"[All Fields] OR "lifestyle"[All Fields] OR "lifestyles"[All Fields]) OR "weight-control"[All Fields] OR ("therapeutics"[MeSH Terms] OR "therapeutics"[All Fields] OR "treatments"[All Fields] OR "therapy"[MeSH Subheading] OR "therapy"[All Fields] OR "treatment"[All Fields] OR "treatment s"[All Fields])) AND ("hasabstract"[All Fields] AND ("case reports"[Publication Type] OR "clinical study"[Publication Type] OR "clinical trial"[Publication Type] OR "clinical trial, phase i"[Publication Type] OR "clinical trial, phase ii"[Publication Type] OR "clinical trial, phase iii"[Publication Type] OR "clinical trial, phase iv"[Publication Type] OR "comparative study"[Publication Type] OR "controlled clinical trial"[Publication Type] OR "government publication"[Publication Type] OR "historical article"[Publication Type] OR "introductory journal article"[Publication Type] OR "journal article"[Publication Type] OR "multicenter study"[Publication Type] OR "observational study"[Publication Type] OR "overall"[Publication Type] OR "pragmatic clinical trial"[Publication Type] OR "randomized controlled trial"[Publication Type] OR "twin study"[Publication Type] OR "validation study"[Publication Type]) AND "humans"[MeSH Terms] AND 1950/01/01:2020/12/31[Date - Publication] AND ("adult"[MeSH Terms] OR "young adult"[MeSH Terms] OR "adult"[MeSH Terms:noexp] OR ("middle aged"[MeSH Terms] OR "aged"[MeSH Terms]) OR "middle aged"[MeSH Terms] OR "aged"[MeSH Terms] OR "aged, 80 and over"[MeSH Terms])) AND ("hasabstract"[All Fields] AND ("case reports"[Publication Type] OR "clinical study"[Publication Type] OR "clinical trial"[Publication Type] OR "clinical trial, phase i"[Publication Type] OR "clinical trial, phase ii"[Publication Type] OR "clinical trial, phase iii"[Publication Type] OR "clinical trial, phase iv"[Publication Type] OR "comparative study"[Publication Type] OR "controlled clinical trial"[Publication Type] OR "government publication"[Publication Type] OR "historical article"[Publication Type] OR "introductory journal article"[Publication Type] OR "journal article"[Publication Type] OR "multicenter study"[Publication Type] OR "observational study"[Publication Type] OR "overall"[Publication Type] OR "pragmatic clinical trial"[Publication Type] OR "randomized controlled trial"[Publication Type] OR "twin study"[Publication Type] OR "validation study"[Publication Type]) AND "humans"[MeSH Terms] AND 1950/01/01:2020/12/31[Date - Publication] AND ("adult"[MeSH Terms] OR "young adult"[MeSH Terms] OR "adult"[MeSH Terms:noexp] OR ("middle aged"[MeSH Terms] OR "aged"[MeSH Terms]) OR "middle aged"[MeSH Terms] OR "aged"[MeSH Terms] OR "aged, 80 and over"[MeSH Terms]))) 6. "exercise"[MeSH Terms] OR "exercise"[All Fields] OR ("physical"[All Fields] AND "activity"[All Fields]) OR "physical activity"[All Fields] OR ("exercise"[MeSH Terms] OR "exercise"[All Fields] OR "exercises"[All Fields] OR "exercise therapy"[MeSH Terms] OR ("exercise"[All Fields] AND "therapy"[All Fields]) OR "exercise therapy"[All Fields] OR "exercise s"[All Fields] OR "exercised"[All Fields] OR "exerciser"[All Fields] OR "exercisers"[All Fields] OR "exercising"[All Fields]) OR ("education"[MeSH Subheading] OR "education"[All Fields] OR "training"[All Fields] OR "education"[MeSH Terms] OR "train"[All Fields] OR "train s"[All Fields] OR "trained"[All Fields] OR "training s"[All Fields] OR "trainings"[All Fields] OR "trains"[All Fields]) 7. ("obeses"[All Fields] OR "obesity"[MeSH Terms] OR "obesity"[All Fields] OR "obese"[All Fields] OR "obesities"[All Fields] OR "obesity s"[All Fields] OR ("overweight"[MeSH Terms] OR "overweight"[All Fields] OR "overweighted"[All Fields] OR "overweightness"[All Fields] OR "overweights"[All Fields]) OR ("metabolic syndrome"[MeSH Terms] OR ("metabolic"[All Fields] AND "syndrome"[All Fields]) OR "metabolic syndrome"[All Fields]) OR "waist-circumference"[All Fields] OR "BMI"[All Fields]) AND ("hasabstract"[All Fields] AND ("case reports"[Publication Type] OR "clinical study"[Publication Type] OR "clinical trial"[Publication Type] OR "clinical trial, phase i"[Publication Type] OR "clinical trial, phase ii"[Publication Type] OR "clinical trial, phase iii"[Publication Type] OR "clinical trial, phase iv"[Publication Type] OR "comparative study"[Publication Type] OR "controlled clinical trial"[Publication Type] OR "government publication"[Publication Type] OR "historical article"[Publication Type] OR "introductory journal article"[Publication Type] OR "journal article"[Publication Type] OR "multicenter study"[Publication Type] OR "observational study"[Publication Type] OR "overall"[Publication Type] OR "pragmatic clinical trial"[Publication Type] OR "randomized controlled trial"[Publication Type] OR "twin study"[Publication Type] OR "validation study"[Publication Type]) AND "humans"[MeSH Terms] AND 1950/01/01:2020/12/31[Date - Publication] AND ("adult"[MeSH Terms] OR "young adult"[MeSH Terms] OR "adult"[MeSH Terms:noexp] OR ("middle aged"[MeSH Terms] OR "aged"[MeSH Terms]) OR "middle aged"[MeSH Terms] OR "aged"[MeSH Terms] OR "aged, 80 and over"[MeSH Terms])) AND (("saudi arabia"[MeSH Terms] OR ("saudi"[All Fields] AND "arabia"[All Fields]) OR "saudi arabia"[All Fields] OR "Gulf"[All Fields] OR "KSA"[All Fields]) AND ("hasabstract"[All Fields] AND ("case reports"[Publication Type] OR "clinical study"[Publication Type] OR "clinical trial"[Publication Type] OR "clinical trial, phase i"[Publication Type] OR "clinical trial, phase ii"[Publication Type] OR "clinical trial, phase iii"[Publication Type] OR "clinical trial, phase iv"[Publication Type] OR "comparative study"[Publication Type] OR "controlled clinical trial"[Publication Type] OR "government publication"[Publication Type] OR "historical article"[Publication Type] OR "introductory journal article"[Publication Type] OR "journal article"[Publication Type] OR "multicenter study"[Publication Type] OR "observational study"[Publication Type] OR "overall"[Publication Type] OR "pragmatic clinical trial"[Publication Type] OR "randomized controlled trial"[Publication Type] OR "twin study"[Publication Type] OR "validation study"[Publication Type]) AND "humans"[MeSH Terms] AND 1950/01/01:2020/12/31[Date - Publication] AND ("adult"[MeSH Terms] OR "young adult"[MeSH Terms] OR "adult"[MeSH Terms:noexp] OR ("middle aged"[MeSH Terms] OR "aged"[MeSH Terms]) OR "middle aged"[MeSH Terms] OR "aged"[MeSH Terms] OR "aged, 80 and over"[MeSH Terms]))) AND ("hasabstract"[All Fields] AND ("case reports"[Publication Type] OR "clinical study"[Publication Type] OR "clinical trial"[Publication Type] OR "clinical trial, phase i"[Publication Type] OR "clinical trial, phase ii"[Publication Type] OR "clinical trial, phase iii"[Publication Type] OR "clinical trial, phase iv"[Publication Type] OR "comparative study"[Publication Type] OR "controlled clinical trial"[Publication Type] OR "government publication"[Publication Type] OR "historical article"[Publication Type] OR "introductory journal article"[Publication Type] OR "journal article"[Publication Type] OR "multicenter study"[Publication Type] OR "observational study"[Publication Type] OR "overall"[Publication Type] OR "pragmatic clinical trial"[Publication Type] OR "randomized controlled trial"[Publication Type] OR "twin study"[Publication Type] OR "validation study"[Publication Type]) AND "humans"[MeSH Terms] AND 1950/01/01:2020/12/31[Date - Publication] AND ("adult"[MeSH Terms] OR "young adult"[MeSH Terms] OR "adult"[MeSH Terms:noexp] OR ("middle aged"[MeSH Terms] OR "aged"[MeSH Terms]) OR "middle aged"[MeSH Terms] OR "aged"[MeSH Terms] OR "aged, 80 and over"[MeSH Terms])) AND (("exercise"[MeSH Terms] OR "exercise"[All Fields] OR ("physical"[All Fields] AND "activity"[All Fields]) OR "physical activity"[All Fields] OR ("exercise"[MeSH Terms] OR "exercise"[All Fields] OR "exercises"[All Fields] OR "exercise therapy"[MeSH Terms] OR ("exercise"[All Fields] AND "therapy"[All Fields]) OR "exercise therapy"[All Fields] OR "exercise s"[All Fields] OR "exercised"[All Fields] OR "exerciser"[All Fields] OR "exercisers"[All Fields] OR "exercising"[All Fields]) OR ("education"[MeSH Subheading] OR "education"[All Fields] OR "training"[All Fields] OR "education"[MeSH Terms] OR "train"[All Fields] OR "train s"[All Fields] OR "trained"[All Fields] OR "training s"[All Fields] OR "trainings"[All Fields] OR "trains"[All Fields])) AND ("hasabstract"[All Fields] AND ("case reports"[Publication Type] OR "clinical study"[Publication Type] OR "clinical trial"[Publication Type] OR "clinical trial, phase i"[Publication Type] OR "clinical trial, phase ii"[Publication Type] OR "clinical trial, phase iii"[Publication Type] OR "clinical trial, phase iv"[Publication Type] OR "comparative study"[Publication Type] OR "controlled clinical trial"[Publication Type] OR "government publication"[Publication Type] OR "historical article"[Publication Type] OR "introductory journal article"[Publication Type] OR "journal article"[Publication Type] OR "multicenter study"[Publication Type] OR "observational study"[Publication Type] OR "overall"[Publication Type] OR "pragmatic clinical trial"[Publication Type] OR "randomized controlled trial"[Publication Type] OR "twin study"[Publication Type] OR "validation study"[Publication Type]) AND "humans"[MeSH Terms] AND 1950/01/01:2020/12/31[Date - Publication] AND ("adult"[MeSH Terms] OR "young adult"[MeSH Terms] OR "adult"[MeSH Terms:noexp] OR ("middle aged"[MeSH Terms] OR "aged"[MeSH Terms]) OR "middle aged"[MeSH Terms] OR "aged"[MeSH Terms] OR "aged, 80 and over"[MeSH Terms]))) 8. (("vision s"[All Fields] OR "vision, ocular"[MeSH Terms] OR ("vision"[All Fields] AND "ocular"[All Fields]) OR "ocular vision"[All Fields] OR "vision"[All Fields] OR "visions"[All Fields] OR "visioning"[All Fields]) AND "2030"[All Fields]) OR "health education"[All Fields] OR "health promotion"[All Fields] OR "prevent*"[All Fields] 9. ("obeses"[All Fields] OR "obesity"[MeSH Terms] OR "obesity"[All Fields] OR "obese"[All Fields] OR "obesities"[All Fields] OR "obesity s"[All Fields] OR ("overweight"[MeSH Terms] OR "overweight"[All Fields] OR "overweighted"[All Fields] OR "overweightness"[All Fields] OR "overweights"[All Fields]) OR ("metabolic syndrome"[MeSH Terms] OR ("metabolic"[All Fields] AND "syndrome"[All Fields]) OR "metabolic syndrome"[All Fields]) OR "waist-circumference"[All Fields] OR "BMI"[All Fields]) AND ("hasabstract"[All Fields] AND ("case reports"[Publication Type] OR "clinical study"[Publication Type] OR "clinical trial"[Publication Type] OR "clinical trial, phase i"[Publication Type] OR "clinical trial, phase ii"[Publication Type] OR "clinical trial, phase iii"[Publication Type] OR "clinical trial, phase iv"[Publication Type] OR "comparative study"[Publication Type] OR "controlled clinical trial"[Publication Type] OR "government publication"[Publication Type] OR "historical article"[Publication Type] OR "introductory journal article"[Publication Type] OR "journal article"[Publication Type] OR "multicenter study"[Publication Type] OR "observational study"[Publication Type] OR "overall"[Publication Type] OR "pragmatic clinical trial"[Publication Type] OR "randomized controlled trial"[Publication Type] OR "twin study"[Publication Type] OR "validation study"[Publication Type]) AND "humans"[MeSH Terms] AND 1950/01/01:2020/12/31[Date - Publication] AND ("adult"[MeSH Terms] OR "young adult"[MeSH Terms] OR "adult"[MeSH Terms:noexp] OR ("middle aged"[MeSH Terms] OR "aged"[MeSH Terms]) OR "middle aged"[MeSH Terms] OR "aged"[MeSH Terms] OR "aged, 80 and over"[MeSH Terms])) AND (("saudi arabia"[MeSH Terms] OR ("saudi"[All Fields] AND "arabia"[All Fields]) OR "saudi arabia"[All Fields] OR "Gulf"[All Fields] OR "KSA"[All Fields]) AND ("hasabstract"[All Fields] AND ("case reports"[Publication Type] OR "clinical study"[Publication Type] OR "clinical trial"[Publication Type] OR "clinical trial, phase i"[Publication Type] OR "clinical trial, phase ii"[Publication Type] OR "clinical trial, phase iii"[Publication Type] OR "clinical trial, phase iv"[Publication Type] OR "comparative study"[Publication Type] OR "controlled clinical trial"[Publication Type] OR "government publication"[Publication Type] OR "historical article"[Publication Type] OR "introductory journal article"[Publication Type] OR "journal article"[Publication Type] OR "multicenter study"[Publication Type] OR "observational study"[Publication Type] OR "overall"[Publication Type] OR "pragmatic clinical trial"[Publication Type] OR "randomized controlled trial"[Publication Type] OR "twin study"[Publication Type] OR "validation study"[Publication Type]) AND "humans"[MeSH Terms] AND 1950/01/01:2020/12/31[Date - Publication] AND ("adult"[MeSH Terms] OR "young adult"[MeSH Terms] OR "adult"[MeSH Terms:noexp] OR ("middle aged"[MeSH Terms] OR "aged"[MeSH Terms]) OR "middle aged"[MeSH Terms] OR "aged"[MeSH Terms] OR "aged, 80 and over"[MeSH Terms]))) AND ("hasabstract"[All Fields] AND ("case reports"[Publication Type] OR "clinical study"[Publication Type] OR "clinical trial"[Publication Type] OR "clinical trial, phase i"[Publication Type] OR "clinical trial, phase ii"[Publication Type] OR "clinical trial, phase iii"[Publication Type] OR "clinical trial, phase iv"[Publication Type] OR "comparative study"[Publication Type] OR "controlled clinical trial"[Publication Type] OR "government publication"[Publication Type] OR "historical article"[Publication Type] OR "introductory journal article"[Publication Type] OR "journal article"[Publication Type] OR "multicenter study"[Publication Type] OR "observational study"[Publication Type] OR "overall"[Publication Type] OR "pragmatic clinical trial"[Publication Type] OR "randomized controlled trial"[Publication Type] OR "twin study"[Publication Type] OR "validation study"[Publication Type]) AND "humans"[MeSH Terms] AND 1950/01/01:2020/12/31[Date - Publication] AND ("adult"[MeSH Terms] OR "young adult"[MeSH Terms] OR "adult"[MeSH Terms:noexp] OR ("middle aged"[MeSH Terms] OR "aged"[MeSH Terms]) OR "middle aged"[MeSH Terms] OR "aged"[MeSH Terms] OR "aged, 80 and over"[MeSH Terms])) AND (((("vision s"[All Fields] OR "vision, ocular"[MeSH Terms] OR ("vision"[All Fields] AND "ocular"[All Fields]) OR "ocular vision"[All Fields] OR "vision"[All Fields] OR "visions"[All Fields] OR "visioning"[All Fields]) AND "2030"[All Fields]) OR "health education"[All Fields] OR "health promotion"[All Fields] OR "prevent*"[All Fields]) AND ("hasabstract"[All Fields] AND ("case reports"[Publication Type] OR "clinical study"[Publication Type] OR "clinical trial"[Publication Type] OR "clinical trial, phase i"[Publication Type] OR "clinical trial, phase ii"[Publication Type] OR "clinical trial, phase iii"[Publication Type] OR "clinical trial, phase iv"[Publication Type] OR "comparative study"[Publication Type] OR "controlled clinical trial"[Publication Type] OR "government publication"[Publication Type] OR "historical article"[Publication Type] OR "introductory journal article"[Publication Type] OR "journal article"[Publication Type] OR "multicenter study"[Publication Type] OR "observational study"[Publication Type] OR "overall"[Publication Type] OR "pragmatic clinical trial"[Publication Type] OR "randomized controlled trial"[Publication Type] OR "twin study"[Publication Type] OR "validation study"[Publication Type]) AND "humans"[MeSH Terms] AND 1950/01/01:2020/12/31[Date - Publication] AND ("adult"[MeSH Terms] OR "young adult"[MeSH Terms] OR "adult"[MeSH Terms:noexp] OR ("middle aged"[MeSH Terms] OR "aged"[MeSH Terms]) OR "middle aged"[MeSH Terms] OR "aged"[MeSH Terms] OR "aged, 80 and over"[MeSH Terms]))) 10. "nutrition s"[All Fields] OR "nutritional status"[MeSH Terms] OR ("nutritional"[All Fields] AND "status"[All Fields]) OR "nutritional status"[All Fields] OR "nutrition"[All Fields] OR "nutritional sciences"[MeSH Terms] OR ("nutritional"[All Fields] AND "sciences"[All Fields]) OR "nutritional sciences"[All Fields] OR "nutritional"[All Fields] OR "nutritionals"[All Fields] OR "nutritions"[All Fields] OR "nutritive"[All Fields] OR "eat"[All Fields] OR "diet*"[All Fields] OR ("food"[MeSH Terms] OR "food"[All Fields]) OR ("feeding"[All Fields] OR "feedings"[All Fields] OR "feeds"[All Fields]) 11. ("obeses"[All Fields] OR "obesity"[MeSH Terms] OR "obesity"[All Fields] OR "obese"[All Fields] OR "obesities"[All Fields] OR "obesity s"[All Fields] OR ("overweight"[MeSH Terms] OR "overweight"[All Fields] OR "overweighted"[All Fields] OR "overweightness"[All Fields] OR "overweights"[All Fields]) OR ("metabolic syndrome"[MeSH Terms] OR ("metabolic"[All Fields] AND "syndrome"[All Fields]) OR "metabolic syndrome"[All Fields]) OR "waist-circumference"[All Fields] OR "BMI"[All Fields]) AND ("hasabstract"[All Fields] AND ("case reports"[Publication Type] OR "clinical study"[Publication Type] OR "clinical trial"[Publication Type] OR "clinical trial, phase i"[Publication Type] OR "clinical trial, phase ii"[Publication Type] OR "clinical trial, phase iii"[Publication Type] OR "clinical trial, phase iv"[Publication Type] OR "comparative study"[Publication Type] OR "controlled clinical trial"[Publication Type] OR "government publication"[Publication Type] OR "historical article"[Publication Type] OR "introductory journal article"[Publication Type] OR "journal article"[Publication Type] OR "multicenter study"[Publication Type] OR "observational study"[Publication Type] OR "overall"[Publication Type] OR "pragmatic clinical trial"[Publication Type] OR "randomized controlled trial"[Publication Type] OR "twin study"[Publication Type] OR "validation study"[Publication Type]) AND "humans"[MeSH Terms] AND 1950/01/01:2020/12/31[Date - Publication] AND ("adult"[MeSH Terms] OR "young adult"[MeSH Terms] OR "adult"[MeSH Terms:noexp] OR ("middle aged"[MeSH Terms] OR "aged"[MeSH Terms]) OR "middle aged"[MeSH Terms] OR "aged"[MeSH Terms] OR "aged, 80 and over"[MeSH Terms])) AND (("saudi arabia"[MeSH Terms] OR ("saudi"[All Fields] AND "arabia"[All Fields]) OR "saudi arabia"[All Fields] OR "Gulf"[All Fields] OR "KSA"[All Fields]) AND ("hasabstract"[All Fields] AND ("case reports"[Publication Type] OR "clinical study"[Publication Type] OR "clinical trial"[Publication Type] OR "clinical trial, phase i"[Publication Type] OR "clinical trial, phase ii"[Publication Type] OR "clinical trial, phase iii"[Publication Type] OR "clinical trial, phase iv"[Publication Type] OR "comparative study"[Publication Type] OR "controlled clinical trial"[Publication Type] OR "government publication"[Publication Type] OR "historical article"[Publication Type] OR "introductory journal article"[Publication Type] OR "journal article"[Publication Type] OR "multicenter study"[Publication Type] OR "observational study"[Publication Type] OR "overall"[Publication Type] OR "pragmatic clinical trial"[Publication Type] OR "randomized controlled trial"[Publication Type] OR "twin study"[Publication Type] OR "validation study"[Publication Type]) AND "humans"[MeSH Terms] AND 1950/01/01:2020/12/31[Date - Publication] AND ("adult"[MeSH Terms] OR "young adult"[MeSH Terms] OR "adult"[MeSH Terms:noexp] OR ("middle aged"[MeSH Terms] OR "aged"[MeSH Terms]) OR "middle aged"[MeSH Terms] OR "aged"[MeSH Terms] OR "aged, 80 and over"[MeSH Terms]))) AND ("hasabstract"[All Fields] AND ("case reports"[Publication Type] OR "clinical study"[Publication Type] OR "clinical trial"[Publication Type] OR "clinical trial, phase i"[Publication Type] OR "clinical trial, phase ii"[Publication Type] OR "clinical trial, phase iii"[Publication Type] OR "clinical trial, phase iv"[Publication Type] OR "comparative study"[Publication Type] OR "controlled clinical trial"[Publication Type] OR "government publication"[Publication Type] OR "historical article"[Publication Type] OR "introductory journal article"[Publication Type] OR "journal article"[Publication Type] OR "multicenter study"[Publication Type] OR "observational study"[Publication Type] OR "overall"[Publication Type] OR "pragmatic clinical trial"[Publication Type] OR "randomized controlled trial"[Publication Type] OR "twin study"[Publication Type] OR "validation study"[Publication Type]) AND "humans"[MeSH Terms] AND 1950/01/01:2020/12/31[Date - Publication] AND ("adult"[MeSH Terms] OR "young adult"[MeSH Terms] OR "adult"[MeSH Terms:noexp] OR ("middle aged"[MeSH Terms] OR "aged"[MeSH Terms]) OR "middle aged"[MeSH Terms] OR "aged"[MeSH Terms] OR "aged, 80 and over"[MeSH Terms])) AND (("nutrition s"[All Fields] OR "nutritional status"[MeSH Terms] OR ("nutritional"[All Fields] AND "status"[All Fields]) OR "nutritional status"[All Fields] OR "nutrition"[All Fields] OR "nutritional sciences"[MeSH Terms] OR ("nutritional"[All Fields] AND "sciences"[All Fields]) OR "nutritional sciences"[All Fields] OR "nutritional"[All Fields] OR "nutritionals"[All Fields] OR "nutritions"[All Fields] OR "nutritive"[All Fields] OR "eat"[All Fields] OR "diet*"[All Fields] OR ("food"[MeSH Terms] OR "food"[All Fields]) OR ("feeding"[All Fields] OR "feedings"[All Fields] OR "feeds"[All Fields])) AND ("hasabstract"[All Fields] AND ("case reports"[Publication Type] OR "clinical study"[Publication Type] OR "clinical trial"[Publication Type] OR "clinical trial, phase i"[Publication Type] OR "clinical trial, phase ii"[Publication Type] OR "clinical trial, phase iii"[Publication Type] OR "clinical trial, phase iv"[Publication Type] OR "comparative study"[Publication Type] OR "controlled clinical trial"[Publication Type] OR "government publication"[Publication Type] OR "historical article"[Publication Type] OR "introductory journal article"[Publication Type] OR "journal article"[Publication Type] OR "multicenter study"[Publication Type] OR "observational study"[Publication Type] OR "overall"[Publication Type] OR "pragmatic clinical trial"[Publication Type] OR "randomized controlled trial"[Publication Type] OR "twin study"[Publication Type] OR "validation study"[Publication Type]) AND "humans"[MeSH Terms] AND 1950/01/01:2020/12/31[Date - Publication] AND ("adult"[MeSH Terms] OR "young adult"[MeSH Terms] OR "adult"[MeSH Terms:noexp] OR ("middle aged"[MeSH Terms] OR "aged"[MeSH Terms]) OR "middle aged"[MeSH Terms] OR "aged"[MeSH Terms] OR "aged, 80 and over"[MeSH Terms]))) 12. "drug therapy"[MeSH Terms] OR ("drug"[All Fields] AND "therapy"[All Fields]) OR "drug therapy"[All Fields] OR "pharmacotherapies"[All Fields] OR "drug therapy"[MeSH Subheading] OR "pharmacotherapy"[All Fields] OR ("drug s"[All Fields] OR "pharmaceutical preparations"[MeSH Terms] OR ("pharmaceutical"[All Fields] AND "preparations"[All Fields]) OR "pharmaceutical preparations"[All Fields] OR "drugs"[All Fields]) OR ("Glucagon-like peptide 1"[MeSH Terms] OR "Glucagon-like peptide 1"[All Fields] OR "glp 1"[All Fields]) OR "Glucagon-like peptide 1"[All Fields] OR ("incretine"[All Fields] OR "incretins"[Pharmacological Action] OR "incretins"[MeSH Terms] OR "incretins"[All Fields] OR "incretin"[All Fields]) OR ("dulaglutide"[Supplementary Concept] OR "dulaglutide"[All Fields]) OR ("exenatide"[MeSH Terms] OR "exenatide"[All Fields] OR "exenatide s"[All Fields]) OR ("semaglutide"[Supplementary Concept] OR "semaglutide"[All Fields]) OR ("liraglutid"[All Fields] OR "liraglutide"[MeSH Terms] OR "liraglutide"[All Fields]) OR ("lixisenatide"[Supplementary Concept] OR "lixisenatide"[All Fields]) OR ("medicin"[All Fields] OR "medicinal"[All Fields] OR "medicinally"[All Fields] OR "medicinals"[All Fields] OR "medicine"[MeSH Terms] OR "medicine"[All Fields] OR "medicine s"[All Fields] OR "medicines"[All Fields]) OR ("liraglutid"[All Fields] OR "liraglutide"[MeSH Terms] OR "liraglutide"[All Fields] OR "saxenda"[All Fields]) OR ("semaglutide"[Supplementary Concept] OR "semaglutide"[All Fields] OR "ozempic"[All Fields]) 13. ("obeses"[All Fields] OR "obesity"[MeSH Terms] OR "obesity"[All Fields] OR "obese"[All Fields] OR "obesities"[All Fields] OR "obesity s"[All Fields] OR ("overweight"[MeSH Terms] OR "overweight"[All Fields] OR "overweighted"[All Fields] OR "overweightness"[All Fields] OR "overweights"[All Fields]) OR ("metabolic syndrome"[MeSH Terms] OR ("metabolic"[All Fields] AND "syndrome"[All Fields]) OR "metabolic syndrome"[All Fields]) OR "waist-circumference"[All Fields] OR "BMI"[All Fields]) AND ("hasabstract"[All Fields] AND ("case reports"[Publication Type] OR "clinical study"[Publication Type] OR "clinical trial"[Publication Type] OR "clinical trial, phase i"[Publication Type] OR "clinical trial, phase ii"[Publication Type] OR "clinical trial, phase iii"[Publication Type] OR "clinical trial, phase iv"[Publication Type] OR "comparative study"[Publication Type] OR "controlled clinical trial"[Publication Type] OR "government publication"[Publication Type] OR "historical article"[Publication Type] OR "introductory journal article"[Publication Type] OR "journal article"[Publication Type] OR "multicenter study"[Publication Type] OR "observational study"[Publication Type] OR "overall"[Publication Type] OR "pragmatic clinical trial"[Publication Type] OR "randomized controlled trial"[Publication Type] OR "twin study"[Publication Type] OR "validation study"[Publication Type]) AND "humans"[MeSH Terms] AND 1950/01/01:2020/12/31[Date - Publication] AND ("adult"[MeSH Terms] OR "young adult"[MeSH Terms] OR "adult"[MeSH Terms:noexp] OR ("middle aged"[MeSH Terms] OR "aged"[MeSH Terms]) OR "middle aged"[MeSH Terms] OR "aged"[MeSH Terms] OR "aged, 80 and over"[MeSH Terms])) AND (("saudi arabia"[MeSH Terms] OR ("saudi"[All Fields] AND "arabia"[All Fields]) OR "saudi arabia"[All Fields] OR "Gulf"[All Fields] OR "KSA"[All Fields]) AND ("hasabstract"[All Fields] AND ("case reports"[Publication Type] OR "clinical study"[Publication Type] OR "clinical trial"[Publication Type] OR "clinical trial, phase i"[Publication Type] OR "clinical trial, phase ii"[Publication Type] OR "clinical trial, phase iii"[Publication Type] OR "clinical trial, phase iv"[Publication Type] OR "comparative study"[Publication Type] OR "controlled clinical trial"[Publication Type] OR "government publication"[Publication Type] OR "historical article"[Publication Type] OR "introductory journal article"[Publication Type] OR "journal article"[Publication Type] OR "multicenter study"[Publication Type] OR "observational study"[Publication Type] OR "overall"[Publication Type] OR "pragmatic clinical trial"[Publication Type] OR "randomized controlled trial"[Publication Type] OR "twin study"[Publication Type] OR "validation study"[Publication Type]) AND "humans"[MeSH Terms] AND 1950/01/01:2020/12/31[Date - Publication] AND ("adult"[MeSH Terms] OR "young adult"[MeSH Terms] OR "adult"[MeSH Terms:noexp] OR ("middle aged"[MeSH Terms] OR "aged"[MeSH Terms]) OR "middle aged"[MeSH Terms] OR "aged"[MeSH Terms] OR "aged, 80 and over"[MeSH Terms]))) AND ("hasabstract"[All Fields] AND ("case reports"[Publication Type] OR "clinical study"[Publication Type] OR "clinical trial"[Publication Type] OR "clinical trial, phase i"[Publication Type] OR "clinical trial, phase ii"[Publication Type] OR "clinical trial, phase iii"[Publication Type] OR "clinical trial, phase iv"[Publication Type] OR "comparative study"[Publication Type] OR "controlled clinical trial"[Publication Type] OR "government publication"[Publication Type] OR "historical article"[Publication Type] OR "introductory journal article"[Publication Type] OR "journal article"[Publication Type] OR "multicenter study"[Publication Type] OR "observational study"[Publication Type] OR "overall"[Publication Type] OR "pragmatic clinical trial"[Publication Type] OR "randomized controlled trial"[Publication Type] OR "twin study"[Publication Type] OR "validation study"[Publication Type]) AND "humans"[MeSH Terms] AND 1950/01/01:2020/12/31[Date - Publication] AND ("adult"[MeSH Terms] OR "young adult"[MeSH Terms] OR "adult"[MeSH Terms:noexp] OR ("middle aged"[MeSH Terms] OR "aged"[MeSH Terms]) OR "middle aged"[MeSH Terms] OR "aged"[MeSH Terms] OR "aged, 80 and over"[MeSH Terms])) AND (("drug therapy"[MeSH Terms] OR ("drug"[All Fields] AND "therapy"[All Fields]) OR "drug therapy"[All Fields] OR "pharmacotherapies"[All Fields] OR "drug therapy"[MeSH Subheading] OR "pharmacotherapy"[All Fields] OR ("drug s"[All Fields] OR "pharmaceutical preparations"[MeSH Terms] OR ("pharmaceutical"[All Fields] AND "preparations"[All Fields]) OR "pharmaceutical preparations"[All Fields] OR "drugs"[All Fields]) OR ("Glucagon-like peptide 1"[MeSH Terms] OR "Glucagon-like peptide 1"[All Fields] OR "glp 1"[All Fields]) OR "Glucagon-like peptide 1"[All Fields] OR ("incretine"[All Fields] OR "incretins"[Pharmacological Action] OR "incretins"[MeSH Terms] OR "incretins"[All Fields] OR "incretin"[All Fields]) OR ("dulaglutide"[Supplementary Concept] OR "dulaglutide"[All Fields]) OR ("exenatide"[MeSH Terms] OR "exenatide"[All Fields] OR "exenatide s"[All Fields]) OR ("semaglutide"[Supplementary Concept] OR "semaglutide"[All Fields]) OR ("liraglutid"[All Fields] OR "liraglutide"[MeSH Terms] OR "liraglutide"[All Fields]) OR ("lixisenatide"[Supplementary Concept] OR "lixisenatide"[All Fields]) OR ("medicin"[All Fields] OR "medicinal"[All Fields] OR "medicinally"[All Fields] OR "medicinals"[All Fields] OR "medicine"[MeSH Terms] OR "medicine"[All Fields] OR "medicine s"[All Fields] OR "medicines"[All Fields]) OR ("liraglutid"[All Fields] OR "liraglutide"[MeSH Terms] OR "liraglutide"[All Fields] OR "saxenda"[All Fields]) OR ("semaglutide"[Supplementary Concept] OR "semaglutide"[All Fields] OR "ozempic"[All Fields])) AND ("hasabstract"[All Fields] AND ("case reports"[Publication Type] OR "clinical study"[Publication Type] OR "clinical trial"[Publication Type] OR "clinical trial, phase i"[Publication Type] OR "clinical trial, phase ii"[Publication Type] OR "clinical trial, phase iii"[Publication Type] OR "clinical trial, phase iv"[Publication Type] OR "comparative study"[Publication Type] OR "controlled clinical trial"[Publication Type] OR "government publication"[Publication Type] OR "historical article"[Publication Type] OR "introductory journal article"[Publication Type] OR "journal article"[Publication Type] OR "multicenter study"[Publication Type] OR "observational study"[Publication Type] OR "overall"[Publication Type] OR "pragmatic clinical trial"[Publication Type] OR "randomized controlled trial"[Publication Type] OR "twin study"[Publication Type] OR "validation study"[Publication Type]) AND "humans"[MeSH Terms] AND 1950/01/01:2020/12/31[Date - Publication] AND AND ("adult"[MeSH Terms] OR "young adult"[MeSH Terms] OR "adult"[MeSH Terms:noexp] OR ("middle aged"[MeSH Terms] OR "aged"[MeSH Terms]) OR "middle aged"[MeSH Terms] OR "aged"[MeSH Terms] OR "aged, 80 and over"[MeSH Terms]))) 14. "bariatric surgery"[MeSH Terms] OR ("bariatric"[All Fields] AND "surgery"[All Fields]) OR "bariatric surgery"[All Fields] OR ("gastroplasty"[MeSH Terms] OR "gastroplasty"[All Fields] OR "gastroplasties"[All Fields]) OR (("sleeve"[All Fields] OR "sleeved"[All Fields] OR "sleeves"[All Fields] OR "sleeving"[All Fields]) AND ("gastrectomy"[MeSH Terms] OR "gastrectomy"[All Fields] OR "gastrectomies"[All Fields])) OR (("gastrics"[All Fields] OR "stomach"[MeSH Terms] OR "stomach"[All Fields] OR "gastric"[All Fields]) AND ("band"[Journal] OR "band"[All Fields])) OR "Roux-en-Y gastric bypass"[All Fields] 15. ("saudi arabia"[MeSH Terms] OR ("saudi"[All Fields] AND "arabia"[All Fields]) OR "saudi arabia"[All Fields] OR "Gulf"[All Fields] OR "KSA"[All Fields]) AND ("hasabstract"[All Fields] AND ("case reports"[Publication Type] OR "clinical study"[Publication Type] OR "clinical trial"[Publication Type] OR "clinical trial, phase i"[Publication Type] OR "clinical trial, phase ii"[Publication Type] OR "clinical trial, phase iii"[Publication Type] OR "clinical trial, phase iv"[Publication Type] OR "comparative study"[Publication Type] OR "controlled clinical trial"[Publication Type] OR "government publication"[Publication Type] OR "historical article"[Publication Type] OR "introductory journal article"[Publication Type] OR "journal article"[Publication Type] OR "multicenter study"[Publication Type] OR "observational study"[Publication Type] OR "overall"[Publication Type] OR "pragmatic clinical trial"[Publication Type] OR "randomized controlled trial"[Publication Type] OR "twin study"[Publication Type] OR "validation study"[Publication Type]) AND "humans"[MeSH Terms] AND 1950/01/01:2020/12/31[Date - Publication] AND ("adult"[MeSH Terms] OR "young adult"[MeSH Terms] OR "adult"[MeSH Terms:noexp] OR ("middle aged"[MeSH Terms] OR "aged"[MeSH Terms]) OR "middle aged"[MeSH Terms] OR "aged"[MeSH Terms] OR "aged, 80 and over"[MeSH Terms])) AND (("bariatric surgery"[MeSH Terms] OR ("bariatric"[All Fields] AND "surgery"[All Fields]) OR "bariatric surgery"[All Fields] OR ("gastroplasty"[MeSH Terms] OR "gastroplasty"[All Fields] OR "gastroplasties"[All Fields]) OR (("sleeve"[All Fields] OR "sleeved"[All Fields] OR "sleeves"[All Fields] OR "sleeving"[All Fields]) AND ("gastrectomy"[MeSH Terms] OR "gastrectomy"[All Fields] OR "gastrectomies"[All Fields])) OR (("gastrics"[All Fields] OR "stomach"[MeSH Terms] OR "stomach"[All Fields] OR "gastric"[All Fields]) AND ("band"[Journal] OR "band"[All Fields])) OR "Roux-en-Y gastric bypass"[All Fields]) AND ("hasabstract"[All Fields] AND ("case reports"[Publication Type] OR "clinical study"[Publication Type] OR "clinical trial"[Publication Type] OR "clinical trial, phase i"[Publication Type] OR "clinical trial, phase ii"[Publication Type] OR "clinical trial, phase iii"[Publication Type] OR "clinical trial, phase iv"[Publication Type] OR "comparative study"[Publication Type] OR "controlled clinical trial"[Publication Type] OR "government publication"[Publication Type] OR "historical article"[Publication Type] OR "introductory journal article"[Publication Type] OR "journal article"[Publication Type] OR "multicenter study"[Publication Type] OR "observational study"[Publication Type] OR "overall"[Publication Type] OR "pragmatic clinical trial"[Publication Type] OR "randomized controlled trial"[Publication Type] OR "twin study"[Publication Type] OR "validation study"[Publication Type]) AND "humans"[MeSH Terms] AND 1950/01/01:2020/12/31[Date - Publication] AND ("adult"[MeSH Terms] OR "young adult"[MeSH Terms] OR "adult"[MeSH Terms:noexp] OR ("middle aged"[MeSH Terms] OR "aged"[MeSH Terms]) OR "middle aged"[MeSH Terms] OR "aged"[MeSH Terms] OR "aged, 80 and over"[MeSH Terms]))) |

Table S2: Search strategy for Ovid

| A) Research theme 1: What are the demographic, cultural and epidemiological factors driving obesity in SA? |
| --- |
| 1. Obesity 2. Obesity, Morbid 3. Obesity, Abdominal 4. Overweight 5. Weight Gain 6. Body mass index 7. BMI 8. obesity.ti 9. obese.ab 10. Body Fat Distribution 11. Adiposity 12. Skinfold thickness 13. waist circumference* 14. waist to hip ratio* 15. Or/1-14 16. Saudi Arabia 17. (Saud* or KSA or Arab* or middle east or MENA or EMRO or orient or arabs or arab or arabia or Gulf) 18. Or/16-17 19. (Risk* OR Epidem* OR behav* OR habit* OR life*style OR prevalence*) 20. (Exercis* OR physical* or active* OR sedentary*) 21. (Food* OR diet* OR eat* OR fe*ding OR nutri*ent Or nutrition*) 22. Or/19-21 23. Adults 24. 15 and 18 and 22 and 23 25. limit to (yr=“1950 -Current”) |
| B) Research theme 2: How are effective treatments for obesity currently being delivered to adults with obesity in SA? |
| 1. Obesity 2. Obesity, Morbid 3. Obesity, Abdominal 4. Overweight 5. Weight Gain 6. Body mass index 7. BMI 8. obesity.ti 9. obese.ab 10. Body Fat Distribution 11. Adiposity 12. Skinfold thickness 13. waist circumference* 14. waist to hip ratio* 15. Or/1-14 16. Saudi Arabia.ti 17. Saudi Arabia.ab 18. (Saud* or KSA or Arab* or middle east or MENA or EMRO or orient or arabs or arab or arabia or Gulf).ti 19. (Saud* or KSA or Arab* or middle east or MENA or EMRO or orient or arabs or arab or arabia or Gulf).ab 20. Or/16-19 21. Intervention 22. Prevention 23. weight management 24. weight control 25. (program* or behav* or lifestyle OR “weight-control” OR treatment* or treat*) 26. (Exercis* OR physical* or active* OR train*ng) 27. Health education 28. Health promotion 29. (Vision 2030 OR “health education” OR “health promotion” OR prevent*) 30. Pharmacotherapy 31. Drugs 32. Medicine 33. Glucagon-like peptide 1 34. GLP-1 35. (incretin or incret* OR dulaglutide OR exenatide OR semaglutide OR liraglutide OR  lirag*tide or lixisenatide OR Saxenda* OR Ozempic*) 36. Bariatric surgery 37. Gastroplasty 38. Gastrectomy 39. Sleeve gastrectomy 40. Gastric band 41. Gastric bypass 42. (bariatric operation OR bariatric operations OR bariatric procedure OR bariatric procedures OR bariatric surgical procedure OR bariatric surgical procedures OR obesity surgery OR sleeve gastrectomy OR sleeve gastrectomy OR gastric sleeve OR biliopancreatic bypass OR biliopancreatic diversion OR duodenal switch OR pancreatobiliary bypass OR gastric banding OR gastric banding OR stomach banding OR swedish gastric banding OR swedish adjustable gastric banding OR laparoscopic adjustable gastric banding OR laparoscopic adjustable silicone banding OR bariatric surgery OR bariatric surgeries OR Gastric bypass OR roux-en-y gastric bypass OR greenville gastric bypass OR gastroileal bypass OR gastrojejunostomy OR gastrojejunostomies OR gastroplasty OR gastroplasties OR collis gastroplasty OR vertical-banded gastroplasty OR vertical banded gastroplasty OR vertical banded gastroplasties OR jejunoileal bypass OR jejuno ileal bypass OR ileojejunal bypass OR ileojejunal bypasses OR intestinal bypass OR intestinal bypasses OR lipectomy OR lipectomies OR aspiration lipectomy OR aspiration lipolysis OR suction lipectomy OR suction lipectomies OR suction lipolysis OR liposuction OR liposuctions OR lipoplasty OR lipoplasties) 43. Or/21-42 44. Adults 45. 15 and 20 and 43 and 44 limit to (yr=“1950 -Current”) |

Table S3: Search strategy for Cochrane

| A) Research theme 1: What are the demographic, cultural and epidemiological factors driving obesity in SA? |
| --- |
| 1. obesity OR overweight OR metabolic syndrome OR "waist-circumference“ OR BMI 2. Saudi Arabia OR Gulf 3. 1 AND 2 4. Risk* OR Epidem* OR behav* OR habit* OR lifestyle OR prevalence 5. 3 AND 4 6. Gene* OR polymorphism OR phenotype OR genome 7. 3 AND 6 8. Exercise OR physical activity OR sedentary 9. 3 AND 8 10. Food OR diet* OR eating OR feeding 11. 3 AND 10 |
| B) Research theme 2: How are effective treatments for obesity currently being delivered to adults with obesity in SA? |
| 1. obesity OR overweight OR metabolic syndrome OR "waist-circumference“ OR BMI 2. Saudi Arabia OR Gulf OR KSA 3. 1 AND 2 4. Intervention* OR “weight management” OR program* OR behav* OR lifestyle OR “weight-control” OR treatment 5. 3 AND 4 6. physical activity OR exercise OR training 7. 3 AND 6 8. Vision 2030 OR “health education” OR “health promotion” OR prevent* 9. 3 AND 8 10. Nutrition OR eating OR diet* OR food 11. 3 AND 10 12. pharmacotherapy OR drugs OR GLP-1 OR “Glucagon-like peptide 1” OR incretin OR dulaglutide OR exenatide OR semaglutide OR liraglutide OR lixisenatide OR medicine OR Saxenda OR Ozempic 13. 3 AND 12 14. bariatric surgery OR gastroplasty OR sleeve gastrectomy OR gastric band OR “Roux-en-Y gastric bypass” 15. 3 AND 13 |

Table S4: Results of systematic literature review for research theme 1

| **Citation** | **Size** | **Study type** | **Age** | **Risk factors of obesity** |
| --- | --- | --- | --- | --- |
| Saudi Med J. 2011 Jun;32(6):621-7. | 312 | Cross sectional | mean age=21.1 | Fat intake, economic status, salt intake |
| J Egypt Public Health Assoc. 2016 Dec;91(4):169-173. doi: 10.1097/01.EPX.0000508457.31670.20. | 401 | Cross-sectional | mean age=28.6 | Not available |
| East Mediterr Health J. 2001 Jul-Sep;7(4-5):716-24. | 810 | Cross-sectional | >65 | Not available |
| J Obes. 2014;2014:961861. doi: 10.1155/2014/961861. | 5000 | Cross-sectional | mean age=43.5 | Not available |
| BMC Womens Health. 2016 Jun 20;16:33. doi: 10.1186/s12905-016-0312-8. | 420 | Cross-sectional | 18-58 | • Being over 35 years old  • Being married • Have at least one child • Education level above high school  • Family income < 10,000 Saudi Riyals (SR)  • Working in the public sector |
| J Obes. 2018 May 21;2018:5246915. doi: 10.1155/2018/5246915. | 907 | Cross-sectional |  | • Age • Siblings with obesity • Parental obesity |
| Mol Biol Rep. 2014 Mar;41(3):1731-40. doi: 10.1007/s11033-014-3022-z. | 2223 | Case control study | >18 | • Variants of SNPs rs7903146, rs1552224 and rs11642841 , rs7903146, rs10440833 |
| Medicine (Baltimore). 2019 Jun;98(23):e15878. doi: 10.1097/MD.0000000000015878. | 200 | Clinical study | 20-50 | • Hypertension • Dyslipidemia • Diabetes mellites • Family history of obesity • Low physical activity • Increased carbohydrate and total fat intake greater than the DRI values |
| Int J Environ Res Public Health. 2020 Jun 13;17(12):4226. doi: 10.3390/ijerph17124226. | 1363 | Cross-sectional | 20-70 | Low fiber intake |
| Lipids Health Dis. 2017 Apr 14;16(1):79. doi: 10.1186/s12944-017-0467-9. | 160 | Cross-sectional | 34.4 ± 8.3 | Not available |
| Lipids Health Dis. 2018 Jun 5;17(1):134. doi: 10.1186/s12944-018-0778-5. | 1019 | Cross-sectional | >18 | Class I obesity • Older age • Being married • High cholesterol • Diabetes Class II/III obesity • Older age • Being civil worker • High cholesterol |
| BMC Public Health. 2020 Aug 8;20(1):1213. doi: 10.1186/s12889-020-09298-w. | 2047 | Cohort |  | Rural residence |
| J Epidemiol Glob Health. 2018 Dec;8(1-2):13-19. doi: 10.2991/j.jegh.2018.09.100. | 454 | Cross-sectional | mean age=20.3 | Overweight/obesity was not associated with physical activity, sedentary behavior, sleeping time, or dietary habits |
| J Adv Nurs. 2018 Dec;74(12):2785-2797. doi: 10.1111/jan.13797. | 299 | Cross-sectional |  | • IPV severity  • Child abuse severity  • Depressive symptoms |
| PLoS One. 2020 Jan 30;15(1):e0228321. doi: 10.1371/journal.pone.0228321. | 10735 | quantitative | >15 | • Television viewing  • Being female  • Increased age • Being married |
| Dis Markers. 2014;2014:758232. doi: 10.1155/2014/758232. | 367 | genotype | 18-30 | rs10767664 (BDNF), rs3751812 (FTO), rs9939609 (FTO), rs9941349 (FTO), rs10938397 (GNPDA2), rs571312 (MC4R), rs2815752 (NEGR1), rs713586 (RBJ), rs543874 (SEC16B), rs7359397 (SH2B1), and rs2867125 (TMEM18) |
| Genet Test Mol Biomarkers. 2017 Jan;21(1):53-57. doi: 10.1089/gtmb.2016.0190. | 396 | case-control, genotype | mean age=24.2 | E4 allele of the APOE gene |
| Mol Biol Rep. 2014 Mar;41(3):1519-23. doi: 10.1007/s11033-013-2997-1. | 204 |  | mean age 23 (male), 21.1 (females) | IL-6 (rs1554606) polymorphism |
| Proceedings of the Nutrition Society, 74(OCE1), E22. doi:10.1017/S0029665115000373 | 601 | Cross-sectional | 19-60 (mean age=29) | Hypertension |
| Saudi Med J. 2012 Dec;33(12):1296-303. | 194 | Cross sectional | mean age=21 | Not available |
| Int J Obes Relat Metab Disord. 2003 Jan;27(1):134-9. doi: 10.1038/sj.ijo.0802181. | 600 | Cross-sectional | 16-84 ,mean age=26 | • Age • Being married |
| Int J Equity Health. 2017 May 30;16(1):90. doi: 10.1186/s12939-017-0588-9. | 10156 | Cross sectional | >=18 | Adverse childhood effects |
| Child Abuse Negl. 2016 Oct;60:10-17. doi: 10.1016/j.chiabu.2016.09.003. | 10156 | Cross sectional | >=18 | Adverse childhood effects |
| J Community Health. 2019 Aug;44(4):815-821. doi: 10.1007/s10900-019-00639-4. | 395 | Cross-sectional | 18-60 | • Being 35 years old or above • Being married • Low physical activity • Unhealthy diet |
| Saudi Med J. 2007 Apr;28(4):559-68. | 17359 | Cross-sectional | 30-70, mean age=46.3 | Inactivity was associated with abdominal obesity |
| Saudi Med J. 2005 May;26(5):824-9. | 17232 | Cross-sectional | 30-70 | • Age • Being female • Urban residence |
| J Community Health. 1997 Jun;22(3):211-23. doi: 10.1023/a:1025177108996. | 10651 | Cross-sectional | >20 | Age, residential area, region, income, gender, and education |
| Int J Obes Relat Metab Disord. 1996 Jun;20(6):547-52. | 13177 | Cross sectional | 15-95 | • Urban residence • Being female • Illiteracy • High income |
| East Mediterr Health J. 2007 Mar-Apr;13(2):441-8. | 17892 | Cross-sectional | 0-100 | • Sedentary life • High calorie intake |
| Biomed Res Int. 2019 Mar 5;2019:8073057. doi: 10.1155/2019/8073057. | 1681 | Cross-sectional | >18 | • Low income • Hypertension |
| Saudi Med J. 2005 Aug;26(8):1260-8. | 1079 | Cross-sectional | >20 | Age |
| The North African J Food Nutrition Res; 2017;1(1):11-18. doi: https://doi.org/10.51745/najfnr.1.01.11-18 | 200 | Cross-sectional | >18 | • Higher daily intake of snacks, dairy products, nuts and grilled food • High fruits intake • High fat intake • Low protein intake • High Dressing intake |
| Nutr J. 2010 Sep 19;9:39. doi: 10.1186/1475-2891-9-39. | 357 | Cross sectional | 18-24 | Not available |
| Hum Genomics. 2013 Jun 5;7(1):15. doi: 10.1186/1479-7364-7-15. | 4650 | case–control study |  | rs6782181_GG |
| Ann Saudi Med. 2017 Mar-Apr;37(2):106-113. doi: 10.5144/0256-4947.2017.106. | 2548 | Cross-sectional | 18-60 | • Being male • Age |
| Saudi Med J. 2013 Apr;34(4):401-7. | 10.229 | cross-sectional | mean age=34.2 | • Age • Education years • Family history of diabetes or hypertension • Eating fruits <2 times a week • Lack of physical activity • Low military rank • Higher education |
| BMJ Open. 2015 Nov 30;5(11):e008590. doi: 10.1136/bmjopen-2015-008590. | 2686 | Cross-sectional |  | • Sleeping >8 hr • Low education • Smoking • Physical inactivity |
| BMC Med Genet. 2018 Nov 20;19(1):203. doi: 10.1186/s12881-018-0715-5. | 493 | Case control study | 18-60 | rs1800592 and rs3811791 of UCP1 gene |
| Genet Test Mol Biomarkers. 2018 Mar;22(3):170-177. doi: 10.1089/gtmb.2017.0218. | 280 | Case control study | mean age:39.78 | rs9939609 and rs1421085 (FTO) |
| Lipids Health Dis. 2018 Mar 27;17(1):58. doi: 10.1186/s12944-018-0679-7. | 329 | case–control study | 18-36 | Trp64Arg polymorphism in ADRB3 gene |
| East Mediterr Health J. 2000 Mar-May;6(2-3):276-82. | 14660 | Cross-sectional | 14-70 | Diabetes |
| BMC Public Health. 2015 Mar 17;15:254. doi: 10.1186/s12889-015-1608-6. | 4758 | Cross-sectional | 15-64 | Being female |
| J Contemp Dent Pract. 2017 Oct 1;18(10):899-904. doi: 10.5005/jp-journals-10024-2146. | 211 | Cross-sectional |  | • Eating outside • Consumption of snacks, cold drinks, and sweets • Not sporting weekly  • Spending >3 hr on TV/Internet |
| J Pak Med Assoc. 2013 Oct;63(10):1285-9. | 428 | Cross sectional | >18, mean age=36.9 | Not available |
| Biomed Environ Sci. 2013 Feb;26(2):94-9. doi: 10.3967/0895-3988.2013.02.003. | 530 | Cross-sectional | 18-72 (mean 36.91 ± 15.22) | Not available |
| J Epidemiol Glob Health. 2018 Dec;8(3-4):183-188. doi: 10.2991/j.jegh.2018.04.100. | 4278 | genotype |  | rs2740434GA |
| J Pak Med Assoc. 2017 Oct;67(10):1541-1546. | 116 | Cross-sectional | 18-26 | • Junk food intake • Use of saturated fat  • Low physical activity |
| Public Health Nutr. 2015 Apr;18(5):784-96. doi: 10.1017/S1368980014001797. | 663 | Cross-sectional | 18-24, mean age=20 | • Being married • Physical inactivity • Having parents with obesity • Having siblings with obesity  • Having highly-educated father  • Intake of French fries/potato chips >3 times/week |
| Saudi Med J. 2007 Dec;28(12):1875-80. | 438 | cross-sectional | 18-60 | • Age  • Obesity • Parity  • Low education |
| Niger J Clin Pract. 2020 Oct;23(10):1356-1367. doi: 10.4103/njcp.njcp_553_19. | 450 | Case control | 18-30 | • Low physical activity  • Increased screen time • Low-education level • Low socioeconomic status • Poor health literacy • Parental negligence |
| Prev Chronic Dis. 2014 Oct 9;11:E174. doi: 10.5888/pcd11.140236. | 10735 | Cross-sectional | >15 | • Age  • Being married • Meat consumption • Diabetes, hypercholesterolemia, hypertension • Primary school educational level or less |
| Obes Facts. 2020;13(1):77-85. doi: 10.1159/000505246. | 3925 | Cross-sectional | >=18 | • Low education • High income • Being financially sponsored by someone else • Being non-single |
| Gene. 2014 Jul 10;544(2):152-8. doi: 10.1016/j.gene.2014.04.064. | 4557 | case–control study | mean age 50 | rs10934857_AA |
| Int J Cardiol. 1997 Dec 19;62(3):227-35. doi: 10.1016/s0167-5273(97)00268-4. | 2049 | Cross sectional |  | Not available |
| Public Health. 1994 Jul;108(4):289-94. doi: 10.1016/s0033-3506(94)80008-1. | 222 | Cross-sectional | 18-25 | Not available |
| Public Health. 1998 Nov;112(6):409-14. doi: 10.1038/sj.ph.1900479. | 144 | Case control study | 15-55 | Not available |
| J Egypt Public Health Assoc. 2007;82(1-2):21-42. | 205 | Cross sectional | mean age=21 | Family history of obesity |
| J Pak Med Assoc. 2017 Mar;67(3):355-359. | 292 | Cross-sectional | 18-24 | • Being male • Having family history of obesity • Having chronic disease |
| PLoS One. 2020 Sep 10;15(9):e0238458. doi: 10.1371/journal.pone.0238458. | 351 | Cross-sectional | 18-26 | • Consumption of carbonated or flavored drinks • Consumption of energy drinks • Eating in front of TV or while playing videogames/ phone • Fast food consumption • Sleep immediately after having dinner • Snacking  • Low physical activity • Eating on dining  • Urban residency • Non-smokers |

Table S5: Results of systematic literature review for research theme 2

| **Citation** | **Study type** | **Sample size** | **Gender** | **Age** | **Intervention implemented** | **Outcomes** |
| --- | --- | --- | --- | --- | --- | --- |
| Afr Health Sci. 2016 Jun;16(2):533-41. doi: 10.4314/ahs.v16i2.22. | RCT | 80 | females | mean age 52.64 | Behavioral lifestyle intervention | BMI reduction, weight loss |
| Afr Health Sci. 2019 Dec;19(4):2881-2891. doi: 10.4314/ahs.v19i4.10. | RCT | 100 | females | 50-58 | Behavioral lifestyle intervention | BMI reduction |
| Eur Neurol. 2019;81(5-6):239-245. doi: 10.1159/000503286. | Retrospective cohort study | 13 | both (77%) | >=18, mean age=26 | sleeve surgery, balloon, and bypass surgery | acute paralytic axonal polyneuropathy (APPAP) |
| Gastrointest Endosc. 2019 Jun;89(6):1132-1138. doi: 10.1016/j.gie.2018.12.012. | case series | 1000 | both (89.7% females) | 18-60 | ESG | %TWL (total weight loss) |
| J Health Psychol. 2014 May;19(5):664-77. doi: 10.1177/1359105313476977. | observational | 7 | females | 26-43 | Bariatric surgery | experiences of obesity, its perceived causes and motives |
| J Obes. 2019 Feb 12;2019:7295978. doi: 10.1155/2019/7295978. | case control | 50 | both (56% females) | mean age=41.4 | Behavioral lifestyle intervention | weight loss |
| Neurosciences (Riyadh). 2016 Jul;21(3):241-5. doi: 10.17712/nsj.2016.3.20160039. | Cross-sectional | 451 | both (69% females) | mean age=37 | bariatric surgery | neurological complications |
| Nutrients. 2018 Mar 20;10(3):383. doi: 10.3390/nu10030383. | RCT | 217 | both | 25-60 | Behavioral lifestyle intervention | Waist circumference reduction |
| Nutrients. 2020 Feb 12;12(2):464. doi: 10.3390/nu12020464. | RCT | 267 | both | >=20 | Behavioral lifestyle intervention | weight loss |
| Obes Facts. 2017;10(5):432-443. doi: 10.1159/000456667. | Retrospective cohort study | 301 | both(67.1 % females) | >=18 | bariatric surgery | readmission rates |
| Obes Surg. 2003 Dec;13(6):918-20. doi: 10.1381/096089203322618777. | Case series | 97 | both (68% female) | mean age=31.7 | SAGB | BMI reduction, weight loss |
| Obes Surg. 2005 Apr;15(4):506-9. doi: 10.1381/0960892053723394. | Case series | 140 | both(62 % females) | 17-53 (mean age=379) | SAGB | BMI reduction, weight loss |
| Obes Surg. 2009 Apr;19(4):456-60. doi: 10.1007/s11695-008-9729-y. | Cohort | 29 |  |  | laparoscopic sleeve gastrectomy | BMI reduction |
| Obes Surg. 2010 Sep;20(9):1219-26. doi: 10.1007/s11695-008-9654-0. | Retrospective cohort study | 173 | both (66.5% females) | mean age=34.5 | IGB | BMI reduction, weight loss |
| Obes Surg. 2017 Aug;27(8):2005-2014. doi: 10.1007/s11695-017-2579-8. | prospective cohort | 100 | both |  | LASG | BMI reduction, weight loss |
| Obes Surg. 2018 Apr;28(4):916-922. doi: 10.1007/s11695-017-2971-4. | Cohort | 213 | both | mean age=36.08 | LSG | BMI reduction, weight loss |
| Obes Surg. 2019 Aug;29(8):2485-2491. doi: 10.1007/s11695-019-03868-7. | case series | 390 | both | mean age=38.6 | LASG, LAGB, LRYGB, LBPD, IGB | Complications |
| Obes Surg. 2019 May;29(5):1694-1696. doi: 10.1007/s11695-019-03796-6. | case series | 10 | both | 23-44 | IGB | Acute Pancreatitis |
| Obes Surg. 2019 Nov;29(11):3547-3552. doi: 10.1007/s11695-019-04024-x. | case series | 20 | both | mean age=40 | revisional sleeve LSG after ESG | % total weight loss (%TWL) |
| Saudi J Gastroenterol. 2017 Mar-Apr;23(2):117-122. doi: 10.4103/1319-3767.203362. | Retrospective cohort study | 108 | both | mean age=35 | Intragastric balloon IGB , IGB with Liraglutide | weight loss, weight regain |
| Saudi J Gastroenterol. 2019 Mar-Apr;25(2):97-100. doi: 10.4103/sjg.SJG_232_18. | Cross-sectional | 624 | both (66% female) | 18-69 | Bariatric surgery | Risks awareness |
| Saudi Med J. 2017 Aug;38(8):837-845. doi: 10.15537/smj.2017.8.20553. | RCT | 140 | both | >20 | Behavioral lifestyle intervention | weight loss |
| Saudi Med J. 2017 Mar;38(3):251-256. doi: 10.15537/smj.2017.3.17033. | case series | 270 | both | mean age:35 | LRYGBP, LSG, LAGB | weight loss |
| Saudi Med J. 2019 Apr;40(4):379-384. doi: 10.15537/smj.2019.4.24050. | Cross-sectional | 1129 | both (65.9% women) | 18-68 | bariatric surgery | public perception of bariatric surgery |
| Surg Laparosc Endosc Percutan Tech. 2017 Dec;27(6):456-459. doi: 10.1097/SLE.0000000000000477. | case study | 4 | both | 24-31 | IGB | acute pancreatitis |
| Clin Med Insights Endocrinol Diabetes. 2019 Mar 18;12:1179551419834935. doi: 10.1177/1179551419834935. | prospective cohort | 64 | both | 30-70 | the addition of liraglutide to the existing treatment | Weight loss |
| J Family Med Prim Care. 2020 Aug 25;9(8):3933-3936. doi: 10.4103/jfmpc.jfmpc_361_20. | Retrospective cohort study | 149 | both |  | Liraglutide | BMI reduction, weight loss |

ESG: Endoscopic sleeve gastroplasty; SAGB: Swedish adjustable gastric band; IGB: intragastric balloon insertion; LSG: laparoscopic sleeve gastrectomy; LAGB: Laparoscopic adjustable gastric banding; LRYGB: Laparoscopic Roux-en-Y gastric bypass; LBPD: Laparoscopic biliopancreatic diversion

Table S6: Risk of bias assessment of RCTs and case-control studies for research theme 2

| **#** | **Study examined (citation)** | **Study type** | **Tool used** | **Overall Risk of bias** |
| --- | --- | --- | --- | --- |
| 1 | Abd El-Kader SM, Saiem Al-Dahr MH. Weight loss improves biomarkers endothelial function and systemic inflammation in obese postmenopausal Saudi women. *Afr Health Sci*. 2016;16(2):533-541. doi:10.4314/ahs.v16i2.22 | RCT | RoB-2 | Some concerns |
| 2 | Wani K, Alfawaz H, Alnaami AM, et al. Effects of A 12-Month Intensive Lifestyle Monitoring Program in Predominantly Overweight/Obese Arab Adults with Prediabetes. *Nutrients*. 2020;12(2):464. Published 2020 Feb 12. doi:10.3390/nu12020464 | RCT | RoB-2 | Low risk |
| 3 | Alghamdi R. Q. (2017). A randomized controlled trial of a 12-week intensive lifestyle intervention program at a primary care obesity clinic for adults in western Saudi Arabia. *Saudi medical journal*, *38*(8), 837–845. https://doi.org/10.15537/smj.2017.8.20553 | RCT | RoB-2 | Low risk |
| 4 | Masood A, Alsheddi L, Alfayadh L, Bukhari B, Elawad R, Alfadda AA. Dietary and Lifestyle Factors Serve as Predictors of Successful Weight Loss Maintenance Postbariatric Surgery. J Obes. 2019;2019:7295978. Published 2019 Feb 12. doi:10.1155/2019/7295978 | Case control | NOS | 6/9 stars |
| 5 | Hakeam HA, O'Regan PJ, Salem AM, Bamehriz FY, Jomaa LF. Inhibition of C-reactive protein in morbidly obese patients after laparoscopic sleeve gastrectomy. *Obes Surg*. 2009;19(4):456-460. doi:10.1007/s11695-008-9729-y | Prospective cohort | NOS | 8/9 but the primary outcome is NOT weight loss |
| 6 | Mosli MM, Elyas M. Does combining liraglutide with intragastric balloon insertion improve sustained weight reduction? [published correction appears in Saudi J Gastroenterol. 2017 May-Jun;23 (3):211]. *Saudi J Gastroenterol*. 2017;23(2):117-122. doi:10.4103/1319-3767.203362 | Case control | NOS | 9/9 |
| 7 | Bawahab MA, Assiri AS, Maksoud WA, et al. Effects of Weight Reduction After Sleeve Gastrectomy on Metabolic Variables in Saudi Obese Subjects in Aseer Province of Kingdom of Saudi Arabia. *Obes Surg*. 2017;27(8):2005-2014. doi:10.1007/s11695-017-2579-8 | before -and-after study | ROBINS | Serious risk |
| 8 | Althuwaini S, Bamehriz F, Aldohayan A, et al. Prevalence and Predictors of Gastroesophageal Reflux Disease After Laparoscopic Sleeve Gastrectomy. Obes Surg. 2018;28(4):916-922. doi:10.1007/s11695-017-2971-4 | Prospective cohort | NOS | 7/9 but the primary outcome is NOT weight loss |
| 9 | AlShareef A, Albaradei O, AlOtaibi HA, Alanazy MH, Abuzinadah AR. Acute Paralytic Post-Bariatric Surgery Axonal Polyneuropathy: Clinical Features and Outcome. Eur Neurol. 2019;81(5-6):239-245. doi:10.1159/000503286 | Retrospective cohort | NOS | 7/9 but the primary outcome is NOT weight loss |
| 10 | Alanazi NK, Ghoraba MA. Effect of Glucagon-like peptide-1 agonist (liriglutide) on weight and glycemic control among adults with type 2 diabetes mellitus attending primary care center at security forces hospital in Riyadh, Saudi Arabia. *J Family Med Prim Care*. 2020;9(8):3933-3936. Published 2020 Aug 25. doi:10.4103/jfmpc.jfmpc_361_20 | Retrospective cohort | NOS | 7/9 but the primary outcome is NOT weight loss |
| 11 | Ahmed A, AlBuraikan D, ALMuqbil B, AlJohi W, Alanazi W, AlRasheed B. Readmissions and Emergency Department Visits after Bariatric Surgery at Saudi Arabian Hospital: The Rates, Reasons, and Risk Factors. *Obes Facts*. 2017;10(5):432-443. doi:10.1159/000456667 | Retrospective cohort study | NOS | 9/9 but the primary outcome is NOT weight loss |
| 12 | Al Hayek AA, Robert AA, Al Dawish MA. Clinical Characteristics and Satisfaction of Liraglutide Treatment among Patients with Type 2 Diabetes: A Prospective Study. *Clin Med Insights Endocrinol Diabetes*. 2019;12:1179551419834935. Published 2019 Mar 18. doi:10.1177/1179551419834935 | before -and-after stud | ROBINS | Serious risk but the primary outcome is NOT weight loss |

Table S7: Risk of bias assessment of observational studies for research theme 2

| **#** | **Study examined (citation)** | **Study type** | **No risk of bias assessed because of** |
| --- | --- | --- | --- |
| 1 | Alqout O, Reynolds F. Experiences of obesity among Saudi Arabian women contemplating bariatric surgery: an interpretative phenomenological analysis. J Health Psychol. 2014;19(5):664-677. doi:10.1177/1359105313476977 | Observational | Study design |
| 2 | Algahtani HA, Khan AS, Khan MA, Aldarmahi AA, Lodhi Y. Neurological complications of bariatric surgery. *Neurosciences (Riyadh)*. 2016;21(3):241-245. doi:10.17712/nsj.2016.3.20160039 | Cross-sectional | Study design |
| 3 | Dhafar KO. Initial experience with Swedish adjustable gastric band at Al-noor hospital. *Obes Surg*. 2003;13(6):918-920. doi:10.1381/096089203322618777 | Case series | Study design |
| 4 | Al-Momen A, El-Mogy I, Ibrahim A. Initial experience with Swedish adjustable gastric band at Saad Specialist Hospital, Al-Khobar, Saudi Arabia. *Obes Surg*. 2005;15(4):506-509. doi:10.1381/0960892053723394 | Case series | Study design |
| 5 | Aljehani Y, AlQattan AS, Alkuwaiti FA, Alsaif F, Aldossari I, Elbawab H. Thoracic Complications of Bariatric Surgeries: Overlooked Entities. *Obes Surg*. 2019;29(8):2485-2491. doi:10.1007/s11695-019-03868-7 | Case series | Study design |
| 6 | Alsohaibani FI, Alkasab M, Abufarhaneh EH, et al. Acute Pancreatitis as a Complication of Intragastric Balloons: a Case Series. *Obes Surg*. 2019;29(5):1694-1696. doi:10.1007/s11695-019-03796-6 | Case series | Study design |
| 7 | Alqahtani AR, Elahmedi M, Alqahtani YA, Al-Darwish A. Laparoscopic Sleeve Gastrectomy After Endoscopic Sleeve Gastroplasty: Technical Aspects and Short-Term Outcomes. *Obes Surg*. 2019;29(11):3547-3552. doi:10.1007/s11695-019-04024-x | Case series | Study design |
| 8 | Alamri AA, Alsadiqi AI, Dahlawi A, et al. Are patients aware of potential risks of weight reduction surgery? An internet based survey. *Saudi J Gastroenterol*. 2019;25(2):97-100. doi:10.4103/sjg.SJG_232_18 | Cross-sectional | Study design |
| 9 | Al Kadi A, Siddiqui ZR, Malik AM, Al Naami M. Comparison of the efficacy of standard bariatric surgical procedures on Saudi population using the bariatric analysis and reporting outcome system. *Saudi Med J*. 2017;38(3):251-256. doi:10.15537/smj.2017.3.17033 | Case series | Study design |
| 10 | Altaf A, Abbas MM. Public perception of bariatric surgery. *Saudi Med J*. 2019;40(4):379-384. doi:10.15537/smj.2019.4.24050 | Cross-sectional | Study design |
